# Fgfr2b signaling is essential for the maintenance of the alveolar epithelial type 2 lineage during lung homeostasis in mice

**DOI:** 10.1007/s00018-022-04327-w

**Published:** 2022-05-19

**Authors:** Negah Ahmadvand, Arun Lingampally, Farhad Khosravi, Ana Ivonne Vazquez-Armendariz, Stefano Rivetti, Matthew R. Jones, Jochen Wilhelm, Susanne Herold, Guillermo Barreto, Janine Koepke, Christos Samakovlis, Gianni Carraro, Jin-San Zhang, Denise Al Alam, Saverio Bellusci

**Affiliations:** 1 Cardio-Pulmonary Institute and Department of Pulmonary and Critical Care Medicine and Infectious Diseases, Universities of Giessen and Marburg Lung Center (UGMLC), Member of the German Center for Lung Research (DZL), Justus-Liebig University Giessen, Giessen, Germany; 2 Department of Physiology, Justus-Liebig University Giessen, Giessen, Germany; 3 Institute for Lung Health, Justus-Liebig University Giessen, Giessen, Germany; 4 Laboratoire IMoPA, UMR 7365 CNRS, Biopole de l’Universite de Lorraine, 54505 Vandoeuvre-les-Nancy, France; 5 Department of Medicine, Cedars-Sinai Medical Center, Lung and Regenerative Medicine Institutes, Los Angeles, CA 90027 USA; 6 The Quzhou Affiliated Hospital of Wenzhou Medical University, Quzhou People’s Hospital, Quzhou, 324000 Zhejiang China; 7 Lundquist Institute for Biomedical Innovation at Harbor-UCLA Medical Center, Los Angeles, CA USA; 8 Laboratory of Extracellular Lung Matrix Remodelling, Department of Internal Medicine, Cardio-Pulmonary Institute and Department of Pulmonary and Critical Care Medicine and Infectious Diseases, Universities of Giessen and Marburg Lung Center (UGMLC), Member of the German Center for Lung Research (DZL), Justus-Liebig University Giessen, Giessen, Germany

**Keywords:** Alveolar epithelial progenitors activation, Amplification and differentiation, Organoids, Lineage tracing, Fgfr2b

## Abstract

Fibroblast growth factor receptor 2b (Fgfr2b) signaling is essential throughout lung development to form the alveolar epithelial lineage. However, its role in alveolar epithelial type 2 cells (AT2s) homeostasis was recently considered dispensable. S*ftpc*^*CreERT2*^;* Fgfr2b*^*flox/flox*^; *tdTomato*^*flox/flox*^ mice were used to delete *Fgfr2b* expression in cells belonging to the AT2 lineage, which contains mature AT2s and a novel Sftpc^Low^ lineage-traced population called “injury activated alveolar progenitors” or IAAPs. Upon continuous tamoxifen exposure for either 1 or 2 weeks to delete *Fgfr2b*, a shrinking of the AT2 population is observed. Mature AT2s exit the cell cycle, undergo apoptosis and fail to form alveolospheres in vitro. However, the lung morphometry appears normal, suggesting the involvement of compensatory mechanisms. In mutant lungs, IAAPs which escaped *Fgfr2b* deletion expand, display enhanced alveolosphere formation in vitro and increase drastically their AT2 signature, suggesting differentiation towards mature AT2s. Interestingly, a significant increase in AT2s and decrease in IAPPs occurs after a 1-week tamoxifen exposure followed by an 8-week chase period. Although mature AT2s partially recover their alveolosphere formation capabilities, the IAAPs no longer display this property. Single-cell RNA seq analysis confirms that AT2s and IAAPs represent stable and distinct cell populations and recapitulate some of their characteristics observed in vivo. Our results underscore the essential role played by Fgfr2b signaling in the maintenance of the AT2 lineage in the adult lung during homeostasis and suggest that the IAAPs could represent a new population of AT2 progenitors.

**Supplementary Information:**

The online version contains supplementary material available at 10.1007/s00018-022-04327-w.

## Introduction

The fibroblast growth factor (Fgf) family is made of 22 members. Fgfs can either act in a paracrine, endocrine, or intracellular fashion. The Fgfs acting through a paracrine mechanism elicit their signaling through fibroblast growth factor receptors (Fgfr) and heparin-sulfate proteoglycans. The endocrine Fgfs signal through Fgfr with the Klotho family of proteins as co-receptors, and the intracellular Fgfs display Fgfr independent signaling [1–3]. The paracrine Fgfs contain Fgf 3, 7, 10, and 22 and interact mainly with Fgfr2b [4]. Among the paracrine Fgfs, Fgf10 takes center stage for its nonredundant role during development, homeostasis, and repair after injury [5–7]. During the pseudoglandular stage of lung development, Fgf10 is expressed dynamically in the mesenchyme in association with the newly formed epithelial buds [8]. Genetic inactivation of *Fgf10* or its receptor *Fgfr2b* leads to a lung displaying the rudimentary primary bronchi, but lacking further ramifications [9–11]. Using an inducible dominant-negative Fgfr2b approach, we characterized both primary transcriptional targets and the main biological activities associated with Fgfr2b signaling. At E12.5, Fgf10 signaling essentially regulates adherens junction and basement membrane organization. Fgf10 acts primarily through beta-catenin signaling and maintains the expression of Sox9, a transcription factor essential for alveolar progenitor differentiation, in the distal epithelium [12]. At E14.5, Fgfr2b signaling controls proliferation of the alveolar epithelial progenitors, and the identified primary transcriptional targets support both overlapping and distinct biological activities as compared to E12.5 [13]. At E16.5, Fgfr2b signaling prevents the differentiation of AT2 progenitors (Jones and Bellusci, unpublished data). Such function is conserved during the alveolar phase of lung development in mice [14]. Fgf10 also plays a vital role during the repair process. For example, *Fgf10* deletion in peribronchial mesenchymal cells leads to impaired repair following injury to the bronchial epithelium using naphthalene [15, 16]. On the other hand, overexpression of *Fgf10* reduces the severity of lung fibrosis in bleomycin-induced mice [17]. Despite these diverse biological activities during development and repair after injury, Fgfr2b signaling in AT2s has been deemed dispensable during homeostasis [14, 18]. Notably, the respective functions of Fgfr2b signaling in our recently described, lineage-traced, AT2 subpopulations has not been defined [19].

Previous studies using the 3D matrigel-based alveolosphere assay in vitro and following diphtheria toxin (DTA)-based genetic deletion of lineage-labeled Sftpc^Pos^ cells using *Sftpc*^*CreERT2/*+^;* Rosa26*^*LSL-DTA/LSL-tdTomato*^ mice in vitro demonstrated the relevance of AT2s as stem cells for the respiratory epithelium [20, 21]. However, in both assays, the self-renewal capability is present only in a subpopulation of lineage-labeled Sftpc^Pos^ AT2s, as only 1–2% of the cultured FACS-isolated lineage-labeled AT2s generated alveolospheres [20]. AT2 stem cells reside in a stromal niche made of lipofibroblasts (LIFs) [5, 20, 22–24]. Some of these LIFs express Fgf10, which acts on the LIFs themselves via Fgfr1b and Fgfr2b to maintain their differentiation [5, 22]. Given the role of Fgf10^Pos^-LIFs in maintaining AT2 stem cell proliferation [24], we propose that Fgf10 signaling to AT2s via Fgfr2b could be instrumental for the maintenance of the AT2 stem cell characteristics.

Using the *Sftpc*^*CreERT2/*+^; *tdtomato*^*flox/flox*^ mice, we previously reported the existence of two distinct AT2 subpopulations called AT2-Tom^Low^ (aka injury-activated alveolar progenitors (IAAPs)) and AT2-Tom^High^ (aka AT2s) [19]. IAAPs express a lower level of *Fgfr2b* and *Etv5*, indicating minor Fgfr2b signaling in these cells and a low level of AT2 differentiation markers *Sftpc*, *Sftpb*, and *Sftpa1.* On the other hand, AT2s show high *Sftpc*, *Sftpb*, and *Sftpa1* and significant activation of Fgfr2 signaling illustrated by the high level of *Fgfr2b* and *Etv5* expression. ATAC-seq analysis indicates these two subpopulations are distinct. Upon pneumonectomy, the number of IAAPs, but not AT2s, increases, and IAAPs display increased expression of *Fgfr2b*, *Etv5*, *Sftpc*, *Ccnd1*, and *Ccnd2* compared to sham. Therefore, our previous work suggested that IAAPs represent quiescent, immature AT2-progenitor cells in mice that could proliferate and differentiate into mature AT2s upon pneumonectomy [19].

The current study analyzes the impact of *Fgfr2b* deletion on AT2s and IAAPs during homeostasis. We have used *Sftpc*^*CreERT2*^; *Fgfr2b*^*flox/flox*^; *tdTomato*^*flox/flox*^ mice to lineage-trace AT2s and IAAPs and delete *Fgfr2b* expression in these subpopulations. In addition, flow cytometry, qPCR, ATAC-seq, gene arrays, scRNA-seq, immunofluorescence, alveolosphere assays, and lung morphometry were carried out. Contrary to the previous studies, our results indicate an essential role for Fgfr2b signaling in IAAPs and AT2s during homeostasis, and help unravel the unexpected behavior of IAAPs, which likely represent a novel AT2 subpopulation with regenerative capabilities.

## Materials and methods

### Animal experiments

All animals were housed under specific pathogen-free (SPF) conditions with free access to food and water. Genetically modified mice including *Sftpc*^*tm1(cre/ERT2*,*rtTA)Hap*^ (stock number 007905), *Fgfr2*^*tm1Dsn*^ (*Fgfr2-IIIb*^*flox*^) [gift from C. Dickson, [4] and the Cre reporter line *tdTomato*^*flox*^ (B6;129S6-Gt(ROSA)26Sor^tm9(CAG-tdTomato)Hze^/J (stock number 007909) were purchased from Jackson Laboratory (Bar Harbor/ME, USA). 8 to 16-week-old mice were treated with tamoxifen‐containing water (1 mg/mL) (T5648, Sigma‐Aldrich, Darmstadt/Germany) to induce Cre recombinase activity. All animal studies were performed according to protocols approved by the Animal Ethics Committee of the Regierungspraesidium Giessen (permit numbers: G7/2017–No.844-GP and G11/2019–No. 931-GP).

### Lung dissociation and FACS

Adult mice were sacrificed, and lungs were perfused with 5 mL PBS through the right ventricle. Next, lungs were inflated via the trachea with dispase and kept in dispase (Coning, NY, USA) and Collagenase Type IV at 37 °C for 40 min with frequent agitation. To obtain single-cell suspensions, the digested tissue was then passed serially through 100-, 70-, and 40-μm cell strainers (BD Biosciences). First, red blood cells (RBC) were eliminated using RBC lysis buffer (Sigma‐Aldrich), according to the manufacturer's protocol. Next, cells were pelleted, resuspended in FACS buffer (0.1% sodium azide, 5% fetal calf serum (FCS), 0,05% in PBS), and stained with antibodies: anti‐EpCAM (APC-Cy7‐conjugated, Biolegend,1:50), CD49F (APC‐conjugated, Biolegend,1:50), anti‐PDPN (FITC‐conjugated, Biolegend, 1:20) and anti-CD274 (unconjugated, Thermo Fisher, 1:100) for 20 min on ice in the dark, followed by washing. Then, the cells were stained with goat antirabbit secondary antibody Alexa flour 488 (Invitrogen, 1:500) for 20 min on ice in the dark, followed by washing. Next, the cells were washed and stained with SYTOX (Invitrogen), a live/dead cell stain according to the manufacturer’s instructions. Finally, flow cytometry data acquisition and cell sorting were carried out using FACSAria III cell sorter (BD Biosciences, San Jose, CA, USA). The data were analyzed using FlowJo software version X (FlowJo, LLC).

### Hematoxylin and eosin staining

Mouse lung tissues were fixed using 4% PFA followed by embedding in paraffin. Paraffin blocks were sectioned into 5-μm-thick slices and placed on glass slides. Following deparaffinization, lung sections were stained with hematoxylin (Roth) for 2 min, washed with running tap water for 10 min, and then stained with eosin (Thermo Fisher Scientific) for 2 min.

### RNA extraction and quantitative real-time PCR

Following lysis of FACS-isolated cells from mouse or human lungs in RLT plus, RNA was extracted using a RNeasy Plus Micro kit (Qiagen), and cDNA synthesis was carried out using QuantiTect reverse transcription kit (Qiagen) according to the manufacturer’s instruction. After that selected primers (Tables 1, 2) were designed via NCBI’s Primer-BLAST option (https://www.ncbi.nlm.nih.gov/tools/primer-blast/) (for primer sequence see supplementary table). Then, quantitative real‐time polymerase chain reaction (qPCR) was performed using PowerUp SYBR Green Master Mix kit according to the manufacturer’s protocol (Applied Biosystems) and run on a LightCycler 480 II machine (Roche Applied Science). *Hypoxanthine guanine phosphoribosyltransferase* (*Hprt*) was used as a mouse reference gene. The data were presented as mean expression relative to *Hprt* and assembled using the GraphPad Prism software (GraphPad Software, La Jolla/CA). Statistical analyses were performed utilizing two tailed-paired Student’s *t* test, and the results were significant when *p* < 0.05.

**Table 1:** qPCR Primer sequences

Primer sequences		
Gene	Forward primer (5′–3′)	Reverse primer (5′–3′)
*Hprt*	CCTAAGATGAGCGCAAGTTGAA	CCACAGGACTAGAACACCTGCTAA
*Fgfr2lllb*	TAAATACGGGCCTGATGGGC	CAGCATCCATCTCCGTCACA
*Etv5*	CAGCCCGCCACGGAG	CCGCTATCACTTTGAAGGGC
*Sftpc*	GGTCCTGATGGAGAGTCCAC	GATGAGAAGGCGTTTGAGG
*Sftpb*	GGCTAGACAGGCAAAAGTGTG	GACCGCGTTCTCAGAGGTG
*Sftpa1*	CAGTGTGATTGGGAGAAACCA	ATGCCAGCAACAACAGTCAA

**Table 2:** Genotyping primer sequences

Genotyping primer sequences		
Gene	Forward primer (5′–3′)	Reverse primer (5′–3′)
*WT*	ATAGGCAGCACCGAGTCCT	ATTCCCCAGCATCCATCTCC
*Fgfr2*	CAGTGGATCAAGCACGTGGA	CTGGCCAAATCTCCAAGGGA

### Immunofluorescence staining

After lung perfusion with PBS through the right ventricle, isolated lungs were fixed with 4% paraformaldehyde. Afterwards, tissues were embedded in paraffin and sectioned at 5 μm thickness. Following deparaffinization, slides were blocked with 3% bovine serum albumin (BSA) (Jackson Immunoresearch Laboratories) in PBS for 1 h at RT. Next, immunofluorescence staining was performed using overnight incubation with polyclonal anti-Prosurfactant Protein C (ProSP-C) (Merck/Millipore/Sigma‐Aldrich, 1:500) followed by staining with polyclonal secondary antibody Goat anti-rabbit Alexa fluor 488 (Invitrogen,1:500). Finally, slides were mounted with ProLong Gold Antifade Reagent containing DAPI (Molecular Probes). Proliferation was assessed using the Click-iT EdU Imaging Kit (Invitrogen, Schwerte, Germany) according to the manufacturer’s instructions. For the EdU experiments, EdU was injected (i.p.) two or 24 h before mice were sacrificed (Dosage: 0.005 mg EdU/g mouse weight). Apoptosis was assessed on paraffin sections or after cytospin via the TdT-mediated dUTP Nick-End Labeling (TUNEL) assay using the DeadEnd Fluorometric TUNEL System (Promega, Walldorf, Germany) according to the manufacturer’s instructions. Apoptosis was quantified by determining the ratio of TUNEL-positive cells to total cells in each region of interest. Multiple images (*n* > 8) were acquired and quantified. For each experiment, sections from at least four independent lungs were analyzed.

### Alveolosphere assay

Sorted epithelial cells (IAAPs/Tom^Low^ and AT2s/Tom^High^) from [*Sftpc*^*CreERT2/*+^; *tdTom*^*flox/flox*^] mice and resident mesenchymal cells from C57BL/6J mice (Epcam^Neg^, Cd31^Neg^, Cd45^Neg^, Sca1^Pos^) were centrifuged and resuspended separately in cell culture medium (Dulbecco’s Modified Eagle Medium, Life Technologies). First, 1 × 10^4^ epithelial cells in 25 μL media and 2 × 10^4^ mesenchymal cells in 25 μL media per insert (12 mm cell culture inserts with 0.4 µm membrane Millipore) were prepared. Next, mesenchymal and epithelial cell suspensions were mixed, followed by the addition of cold Matrigel^®^ growth factor-reduced Matrigel (Corning) at a 1:1 dilution resulting in 100 μL final volume per insert. Then, Matrigel cell suspensions were placed on the top of the filter membrane of the insert and incubated at 37 °C for 5 min. Next, 350 μL of the medium was transferred to each well. Finally, cells were incubated in air–liquid interface conditions at 37 °C with 5% CO_2_ for 2 weeks. Media were changed 3 times per week.

### Microarray

Purified total RNA was amplified using the Ovation PicoSL WTA System V2 kit (NuGEN Technologies). Per sample, 2 µg amplified cDNA was Cy5-labeled using the SureTag DNA labeling kit (Agilent). Hybridization to 8 × 60K 60mer oligonucleotide spotted microarray slides (Human Mouse Genome, Agilent Technologies, design ID 074809) and subsequent washing and drying of the slides were performed following the Agilent hybridization protocol in Agilent hybridization chambers, with following modifications: 3 µg of the labeled cDNA was hybridized for 22 h at 65 °C. The cDNA was not fragmented before hybridization.

The dried slides were scanned at 2 µm/pixel resolution using the InnoScan is900 (Innopsys). Image analysis was performed with Mapix 6.5.0 software, and calculated values for all spots were saved as GenePix results files. Stored data were evaluated using the R software and the limma package from BioConductor. Log2 mean spot signals were taken for further analysis. The data were background corrected using the NormExp procedure on the negative control spots and quantile-normalized before averaging. Log2 signals of replicate spots were averaged, and from several different probes addressing the same gene, only the probe with the highest average signal was used. Genes were ranked for differential expression using a moderated *t* statistic. Finally, pathway analyses were done using gene set tests on the ranks of the *t* values. Pathways were taken from the KEGG database (http://www.genome.jp/kegg/pathway.html). The raw data have been deposited in GEO (accession number GSE162588).

### ATAC-seq

25,000 FACS-sorted cells were collected and used for ATAC Library preparation using Tn5 Transposase from Nextera DNA Sample Preparation Kit (Illumina). The cell pellet was resuspended in 50 µL Lysis/Transposition reaction (12.5 µL THS-TD-Buffer, 2.5 µL Tn5, 5 µL 0.1% Digitonin, and 30 µL water) and incubated at 37 °C for 30 min with occasional snap mixing. Following purification of the DNA, fragments were done by Min Elute PCR Purification Kit (Qiagen). Amplification of the Library together with Indexing Primers was performed as described. Libraries were mixed in equimolar ratios and sequenced on the NextSeq500 platform using V2 chemistry. Trimmomatic version 0.38 was employed to trim reads after a quality drop below a mean of Q15 in a window of 5 nucleotides. Only reads longer than 15 nucleotides were cleared for further analyses. Trimmed and filtered reads were aligned versus the mouse genome version mm10 (GRCm38) using STAR 2.6.1d with the parameters “--outFilterMismatchNoverLmax 0.1 --outFilterMatchNmin 20 --alignIntronMax 1 --alignSJDBoverhangMin 999 --outFilterMultimapNmax 1 --alignEndsProtrude 10 ConcordantPair” and retaining unique alignments to exclude reads of uncertain origin. Reads were further deduplicated using Picard 2.18.16 (Picard: A set of tools (in Java) for working with next-generation sequencing data in the BAM format) to mitigate PCR artefacts leading to multiple copies of the same original fragment. Reads aligning to the mitochondrial chromosome were removed. The Macs2 peak caller version 2.1.2 was employed to accommodate the range of peak widths typically expected for ATAC-seq. The minimum *q* value was set to − 4, and FDR was changed to 0.0001. Peaks overlapping ENCODE blacklisted regions (known misassemblies, satellite repeats) were excluded.

To be able to compare peaks in different samples to assess reproducibility, the resulting lists of significant peaks were overlapped and unified to represent identical regions. Sample counts for union peaks were produced using bigWigAverageOverBed (UCSC Toolkit) and normalized with DESeq2 1.18.1 to compensate for differences in sequencing depth, library composition, and ATAC-seq efficiency. Peaks were annotated with the promoter of the nearest gene in range (TSS ± 5000 nt) based on reference data of GENCODE vM15.

### scRNA-seq

Single-cell suspensions were processed using the 10 × Genomics Single Cell 3′ v3 RNA-seq kit. Gene expression libraries were prepared according to the manufacturer’s protocol. In addition, MULTI-seq barcode libraries were retrieved from the samples and libraries were prepared independently.

### Sequencing and processing of raw sequencing reads

Sequencing was done on Nextseq2000, and raw reads were aligned against the mouse genome (mm10, ensemble assembly 104) and mapped and counted by StarSolo (Dobin et al., 10.1093/bioinformatics/bts635) followed by secondary analysis in Annotated Data Format. Pre-processed counts were analyzed using Scanpy (Wolf et al., 10.1186/s13059-017-1382-0). Basic cell quality control was conducted by considering the number of detected genes and mitochondrial content. Cells expressing less than 300 genes or having a mitochondrial content of more than 20% were removed from the analysis. Further, we filtered genes if detected in less than 30 cells (< 3%). Raw counts per cell were normalized to the median count over all cells and transformed into log space to stabilize the variance. We initially reduced the dimensionality of the dataset using PCA, retaining 50 principal components. Subsequent steps, like low-dimensional UMAP embedding (McInnes and Healy, https://arxiv.org/abs/1802.03426) and cell clustering via community detection (Traag et al., https://arxiv.org/abs/1810.08473), were based on the initial PCA. Final data visualization was done by the cellxgene package. The raw data have been deposited in GEO (accession number GSE199112).

## Results

### IAAP and AT2 subpopulations respond differently to *Fgfr2b* deletion

We recently isolated two AT2 lineage-labeled Sftpc^Pos^ cells (called IAAPs and mature AT2s) in the mouse adult lung based on the differential levels of Tomato expression downstream of the *Rosa26* promoter [19]. A common assumption in the field is that the *Rosa26* locus is ubiquitous (expressed in all the cells) and homogeneous (expressed at the same level in all the cells of the body). Against these assumptions, previous analysis of *Rosa26*-*LacZ* mice had established that LacZ expression was not uniform throughout the embryo [25]. In addition, LacZ expression was also described to be heterogeneous in the adult mouse [Jackson lab (129-*Gt(ROSA)26Sor*/J Stock No: 002292)]. Interestingly, IAAPs and AT2s were observed regardless of whether *tdTomato*^*flox/flox*^ or *tdTomato*^*flox/*+^ reporter mice were used [19]. These results support the conclusion that it is primarily the level of tomato expression from the *Rosa26* promoter and not the efficiency of recombination of the *LoxP-STOP-LoxP-tomato* cassette downstream of the *Rosa26* promoter, which is different in these two subpopulations. We also found that IAAPs expressed programmed death ligand 1 (Pd-l1), a cell surface immune checkpoint inhibitor which was initially described in the context of cancer cells (Fig. S1).

**Fig. 1:**
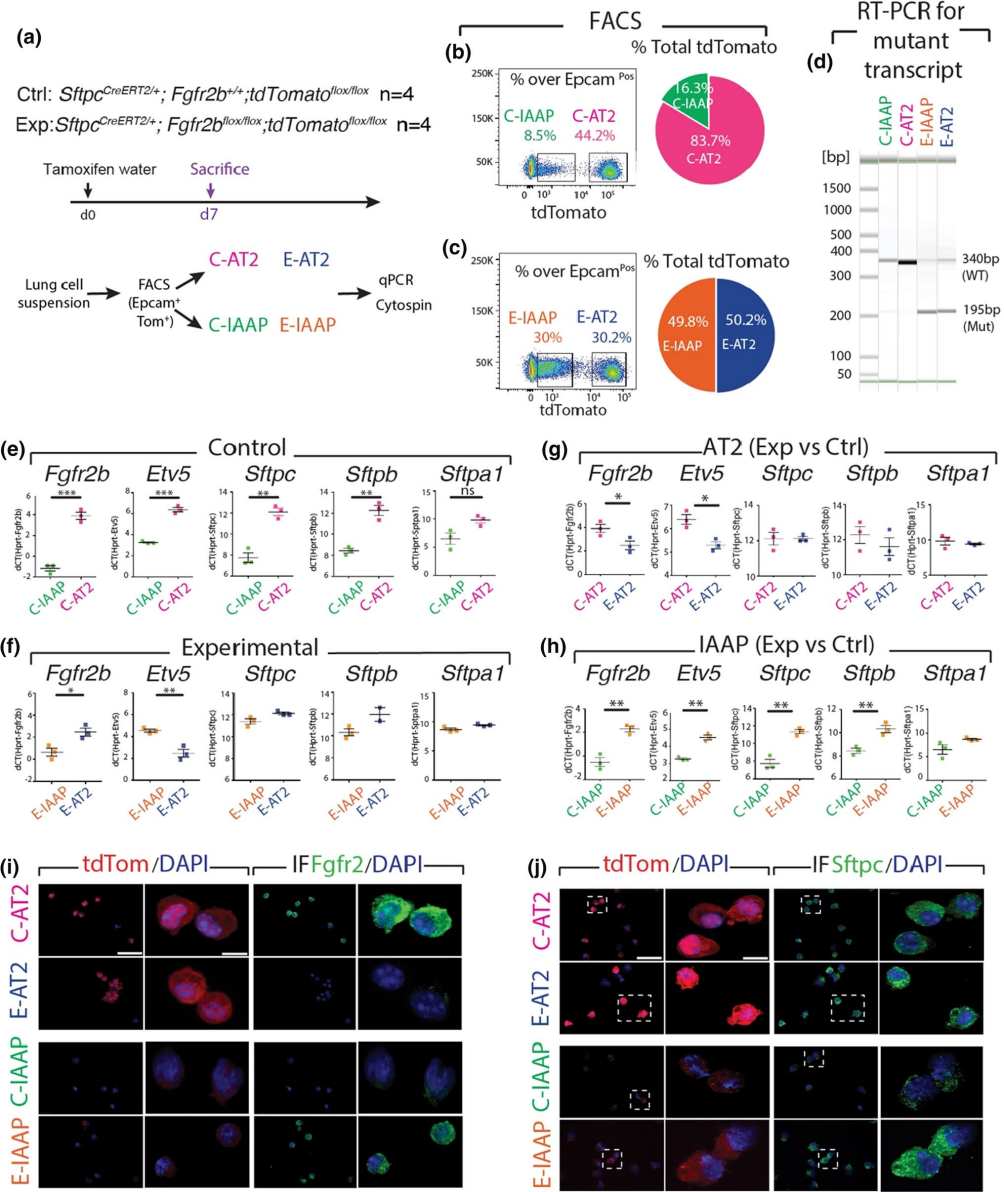
IAAP and AT2 subpopulations respond differently to *Fgfr2b* deletion. **a** Timeline of tamoxifen treatment of *Sftpc*^*CreERT2/*+^; *Fgfr2b*^+*/*+^; *tdTom*^*flox/flox*^ control mice and *Sftpc*^*CreERT2/*+^; *Fgfr2b*^*flox/flox*^; *tdTom*^*flox/flox*^ experimental mice (*n* = 4). **b**,** c** Representative flow cytometry plots represent the detection of IAAP and AT2 subpopulations based on the tdTomato level in control and experimental lungs. The pie chart shows the percentage of IAAPs and AT2s in a representative flow cytometry experiment in total tdTomato positive cells in the control and experimental groups. **d** RT-PCR for detecting the *Fgfr2b* mutant transcript in FACS-based sorted C-IAAPs, C-AT2s, E-IAAPs and E-AT2s. Wild type and mutant forms are detected by the size of 340 bp and 195 bp, respectively. **e** qPCR analysis of FACS-based sorted C-IAAPs and C-AT2s. **f** qPCR analysis of FACS-based sorted E-IAAPs and E-AT2s. **g** qPCR gene expression analysis of FACS-based sorted C-AT2s and E-AT2s. **h** qPCR gene expression analysis of FACS-based sorted C-IAAPs and E-IAAPs. **i** Immunofluorescence staining against Fgfr2 on cytospins of C-AT2s, E-AT2s, C-IAAPs and E-IAAPs (Scale bar: 50 μm). **j** Sftpc immunofluorescen staining on cytospins of C-AT2s, E-AT2s, C-IAAPs, and E-IAAPs (*n* = 4) (Scale bar: 50 μm). The data are presented as mean values ± SEM. *p < 0.05, **p < 0.01, ***p < 0.001

To unravel the function of Fgfr2b signaling in the AT2 lineage, *Sftpc*^*CreERT2/*+^; *Fgfr2b*^*flox/flox*^; *tdTom*^*flox/flox*^ (*Fgfr2b-*cKO) mice, called the experimental (Exp.) group, were initially treated with tamoxifen water for 7 days. *Sftpc*^*CreERT2/*+^; *Fgfr2b*^+*/*+^; *tdTom*^*flox/flox*^ mice undergoing the same treatment were used as controls (Ctrl.) (Fig. 1a).

Flow cytometry analysis on harvested lungs underpinned the presence of IAAP (AT2-Tom^Low^) and AT2 (AT2-Tom^High^) populations. We named the IAAP and AT2 cells from the Ctrl. animals, C-IAAPs and C-AT2s, respectively, and the IAAP and AT2 cells from the Exp. animals, E-IAAPs and E-AT2s, respectively. In Ctrl. lungs, we observed an average of 9.93 ± 1.13% (*n* = 4) C-IAAPs and 44.75% ± 1.22% (*n* = 4) C-AT2s (of total Epcam^Pos^), as previously described (Fig. 1b) [19]. In Exp. lungs, we found that E-IAAPs represented 27.05% (27.05 ± 1.83%, *n* = 4) of the overall Epcam^Pos^ cells, and the E-AT2s represented 25.70% (25.70 ± 2.45%, *n* = 4) of the overall Epcam^Pos^ cells (Fig. 1c). The decrease in the number of AT2 cells in Exp. vs Ctrl. lungs (25.70% vs 44.75%, respectively) indicates that the Fgfr2b pathway is critical for maintaining AT2s. Interestingly, a concomitant increase in the percentage of IAAPs in Exp. vs Ctrl. was observed (27.05% vs 9.93%), suggesting that IAAPs, previously quiescent, were becoming active and proliferative.

Next, we examined whether *Fgfr2b* was haplosufficient in the alveolar epithelial lineage by investigating the consequences of losing a single copy of *Fgf2b* on the percentage of AT2s and IAAPs. Using *Fgfr2b* heterozygous (het.) mice and Ctrl. *Fgf2b*^+*/*+^ (WT) mice, we carried out the previously described FACS-based approach to isolate AT2s and IAAPs [19]. No difference in the percentage of IAAPs and AT2s in WT (*Fgfr2b*^+*/*+^) vs *Fgfr2b*^+/−^ hets. could be detected, indicating that *Fgfr2b* is haplosufficient in Sftpc-expressing cells (Fig. S2).

To investigate the efficiency of recombination in the IAAPs and AT2s in Exp. (*Sftpc*^*CreERT2/*+^;* dTomato*^*flox/flox*^;* Fgfr2b*^*flox/flox*^) (*n* = 3) vs Ctrl. lungs (*Sftpc*^*CreERT2/*+^;* dTomato*^*flox/flox*^;* Fgfr2b*^+*/*+^) (*n* = 2) (Fig. S3), we analyzed the lungs 36 h after a single dose of Tam IP. FACS analysis was carried out to quantify the abundance of IAAPs and AT2s (out of Epcam) in Ctrl. and Exp. lungs. We chose the early 36-h time point to analyze the recombination events before the possible onset of a phenotype linked to *Fgfr2b* deletion, which could impact the IAAP/AT2 ratio. As a quality control, we found a similar percentage of Epcam positive cells over total cells in Exp. vs Ctrl. (23.4% vs 21.1%, respectively) (Fig. 3a). Next, we analyzed the percentage of AT2s, which at later time points (at day 7 and 14 on continuous Tam water) was significantly decreased in Exp. vs Ctrl. lungs (Figs. 1b, c and 4b). As we used *tdTomato*^*flox/flox*^ mice, we observed two peaks for the AT2s corresponding to one vs. two copies of the recombined *LoxP-STOP-LoxP-tomato* cassette. We found a slightly higher percentage of AT2s over Epcam in Exp. vs Ctrl. lungs (37.7% vs 29.4%, respectively), indicating that the efficiency of labeling in AT2s in Exp. lungs was not impaired compared to Ctrl. lungs. The percentage of IAAPs over Epcam^Pos^ cells in Exp. vs Ctrl. lungs (6.3% vs 7.6%, respectively) indicate similar labeling of these cells in Ctrl. and Exp. conditions (Fig. 3a). Altogether, these data indicate that the efficiency of labeling of either IAAPs or AT2s in Ctrl. or in Exp. lungs is not significantly altered in either condition. This suggests that the increase in the IAAPs to AT2 ratio in Exp. lungs at later time points is not due to a difference in the recombination efficiency of the *Rosa26* locus at earlier time points. We also compared the global efficiency of recombination at later time points in Exp. vs Ctrl. by IF (without distinguishing between IAAPs and AT2s, as this is not possible by IF using only Tomato) by quantifying the percentile of Tom^Pos^Sftpc^Pos^/Sftpc^Pos^ at days 7 and 14. Our results at day 7 show similar proportions of Tom^Pos^Sftpc^Pos^/Sftpc^Pos^ (d7: 77% ± 5.4 in Ctrl. vs 70% ± 0.48 in Exp., *n* = 4). A similar observation was also made at day 14 (d14: 84% ± 4.23 in Ctrl. vs 82% ± 3.97 in Exp., *n* = 4) (Fig. S3b).

To investigate whether *Fgfr2b* was successfully deleted in both IAAPs and AT2s, RT-PCR was carried out to detect the wild type and mutant *Fgfr2b* transcripts (Fig. 1d) [4]. The mutant *Fgfr2b* transcript (195 bp) was present in E-IAAPs and E-AT2s in *Fgfr2b-*cKO lungs, and as expected, was not detected in the corresponding cells (C-IAAPs and C-AT2s) from Ctrl. lungs (Fig. 1d). Sequencing of wild type and mutant cDNA bands that were cut and purified from agarose gel confirmed the deletion of exon 8 encoding the *Fgfr2b* isoform in the mutant cDNA (Fig. S4b).

Next, qPCR was performed on FACS-isolated IAAPs and AT2s isolated from Ctrl. and Exp. lungs. We investigated genes which are downstream of Fgfr2b signaling. As previously described, we found that C-AT2s as compared to C-IAAPs were enriched in *Fgfr2b*, *Etv5*, and the differentiation markers *Sftpc*, *Sftpb*, and *Sftpa1*, indicating higher level of Fgfr2b signaling in C-AT2s vs C-IAAPs (Fig. 1e) [19]. However, in contrast to the Ctrl., the difference in *Fgfr2b* expression between E-IAAPs and E-AT2s was reduced. Moreover, *Etv5* expression was significantly downregulated in E-AT2s vs E-IAAPs, and the expression levels of *Sftpc*,* Sftpb*, and *Sftpa1* were not substantially different between E-IAAPs and E-AT2s (Fig. 1f) indicating decreased Fgfr2b signaling in E-AT2s vs E-IAAPs.

### Fgfr2b inactivation in the AT2 lineage leads to the loss of Fgfr2b signaling in AT2s and activation of Fgfr2b signaling in IAAPs

The AT2s and IAAPs were compared between Exp. and Ctrl. lungs using qPCR and immunofluorescence staining on cytospins of sorted cells (Fig. 1a). qPCR analysis of AT2 demonstrated a significant decrease of *Fgfr2b* and *Etv5* expressions in Exp. vs Ctrl. lungs, corroborating the loss of Fgfr2b signaling in these cells; however, no changes in the expression of *Sftpc*,* Sftpb*, and *Sftpa1* were observed in these cells (Fig. 1g). In contrast, in IAAPs, significant upregulation of *Fgfr2b*, *Etv5*,* Sftpc*, and *Sftpb* was identified (Fig. 1h). These changes in *Fgfr2b* and *Sftpc* mRNA levels were validated at the protein level by cytospin of isolated AT2s and IAAPs from Exp. and Ctrl. lungs, followed by immunofluorescence staining (Fig. 1i, j). These results support the loss of Fgfr2b signaling in AT2s and activation of Fgfr2b signaling in IAAPs in Exp. vs Ctrl. lungs.

### ATAC-seq analysis and transcriptomic analyses reveal that *Fgfr2b* deletion leads to activation of IAAP cells

To carry out genome-wide profiling of the epigenomic landscape, an assay for transposase-accessible chromatin using sequencing (ATAC-seq) was performed on C-IAAP and E-IAAP subpopulations at day 7 on tamoxifen water (Fig. S5). Interestingly, our data indicate a high signal background in E-IAAPs (data not shown), often seen in dying cells [26].

After correcting this elevated background to remove the contribution of dying cells, common and distinct peaks were identified for C-IAAPs and E-IAAPs (Fig. S5a, b). Gene set enrichments based on the regions of opened chromatin were carried out. Gene set enrichment (corrected P value smaller than 0.2, top 50 sets) between C-IAAPs and E-IAAPs using Kobas PANTHER predicted that genes belonging to the inflammation mediated by chemokines and cytokine signaling pathway as well as genes controlling apoptosis signaling pathways were significantly upregulated in E-IAAPs compared to C-IAAPs (data not shown). Further analysis of the ATAC-seq data using the Reactome database indicated that the chromatin in loci of genes belonging to metabolism, metabolism of lipids and lipoprotein and immune genes was more open in E-IAAPs (Fig. S5c).

**Fig. 2:**
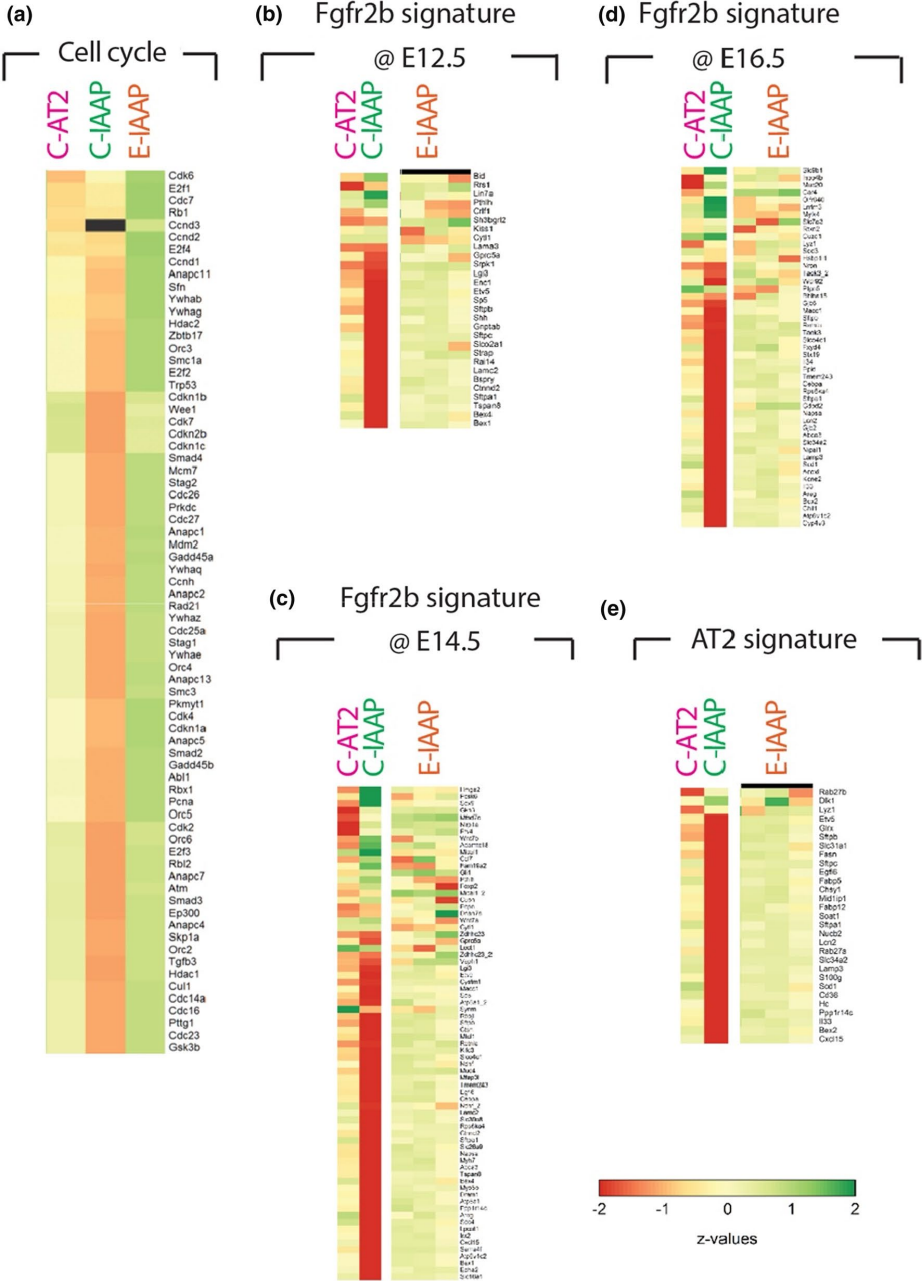
Gene arrays comparing C-AT2s, C-IAAPs, and E-IAAPs. **a** Heatmap for the cell cycle genes indicating up-regulation of cell cycle genes in E-IAAP. **b**–**d** Heatmap for the Fgfr2b signature at E12.5, E14.5, and E16.5 indicating increased Fgfr2b signaling in E-IAAPs. **e** Heatmap for the AT2 signature supporting the increased commitment of the E-IAAPs towards the AT2 lineage

We also explored, using gene array carried out between C-AT2s, C-IAAPs and E-IAAPs captured at day 7 of Tam water exposure, the status of genes belonging to cell cycle regulation, to the Fgfr2b transcriptomic signature previously identified [12, 13, 27], and to the AT2 signature [28] (Fig. 2). Our data confirm that C-IAAPs are quiescent cells as compared to C-AT2s. However, we observed a drastic upregulation of cell cycle genes in E-IAAPs, consistent with their activated status (Fig. 2a). We also found that Fgfr2b signature genes at E12.5 [12] (Fig. 2b), E14.5 [13] (Fig. 2c), and E16.5 [27] (Fig. 2d) were enriched in C-AT2s vs C-IAAPs. These signatures were upregulated in E-IAAPs, supporting the activation of Fgfr2b signaling in these cells (Fig. 2b–d). Finally, we examined the status of the AT2 signature. As previously described, the C-AT2s are mature AT2s and express a high level of the AT2 signature as compared to the undifferentiated C-IAAPs. In contrast, E-IAAPs display a significant enrichment in the AT2 signature, suggesting that these cells differentiate towards mature AT2s (Fig. 2e).

Altogether, we conclude that the E-IAAPs analyzed on day 7 during tamoxifen water exposure are made of apoptotic and surviving cells. After background correction, a sub-population of E-IAAPs emerges, which is highly active metabolically and likely proliferative, and which displays increased Fgfr2b signaling along with enhanced AT2 signature expression. This subpopulation appears to be geared towards lipoprotein metabolism, which is associated with surfactant production.

### Genomic analysis in *Fgfr2b-cKO* reveals that the *Fgfr2b* locus is differentially impacted between mature AT2s and IAAPs

**Fig. 3:**
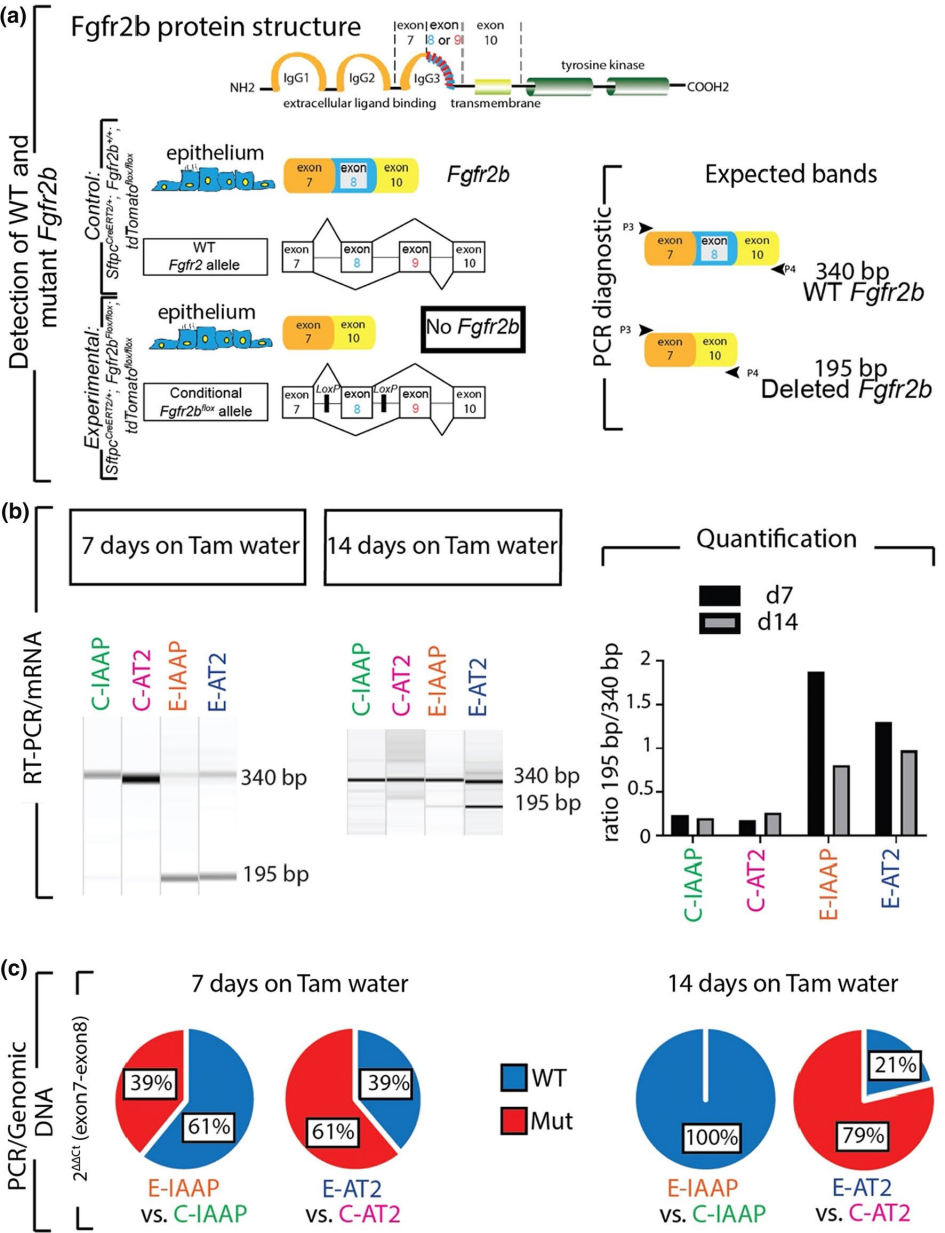
Continuous deletion of the *Fgfr2b* allele in AT2 cells and amplification of IAAP cells. **a** Schematic of Fgfr2b protein structure, coding mRNA, and DNA. Wild-type *Fgfr2b* transcript consists of exon 7, exon 8, and exon 10, which is detected by the band size of 340 bp, and mutant *Fgfr2b* form (exon 8 deleted) is detectable by the band size of 195 bp. **b** RT-PCR for detecting WT and *Fgfr2b* mutant transcripts in FACS-based sorted C-IAAPs, C-AT2s, E-IAAPs, and E-AT2s on day 7 and day 14. Quantification of the corresponding PCR bands. **c** Pie charts represent qPCR data for deleted exon 8 (mutated Fgfr2b locus) vs exon 7 (reflecting the intact *Fgfr2* locus) to detect the relative extent of the mutated and wild-type *Fgfr2b* locus in E-IAAPs vs C-IAAPs and E-AT2s vs C-AT2s at two time points

Given the surprising result that Fgfr2b signaling was activated in E-IAAPs despite the *Fgfr2b* deletion observed initially in these cells on day 7 after tamoxifen treatment (Fig. 1e–h), the mice were treated for a longer time with tamoxifen to ensure that *Fgfr2b* deletion was complete. Next, the presence of the mutant and wild-type *Fgfr2b* transcripts was analyzed by RT-PCR on day 14 after tamoxifen treatment (continuous tamoxifen water treatment). Surprisingly, the results indicate that in E-IAAPs from *Fgfr2b-cKO* lungs, the mutated *Fgfr2b* transcript was barely detectable, while the mutated transcript was still detected in *Fgfr2b-cKO* E-AT2s at both time points (Fig. 3a, b).

To quantify the mutated and wild type *Fgfr2b* on day 7 and day 14, qPCR for the detection of exon 8 (the deleted exon) vs exon 7 (reflecting the intact *Fgfr2b* locus) was performed on the genomic DNA of AT2s and IAAPs from *Fgfr2b-cKO* and Ctrl. lungs. The results show that on day 7, the relative presence of the mutated and wild-type *Fgfr2b* in E-IAAPs was 39% and 61%, respectively. However, on day 14, wild-type *Fgfr2b* increased to 100% while the mutated *Fgfr2b* was no longer detected, indicating that at this time point E-IAAPs contained mostly the wild-type *Fgfr2b* (Fig. 3c). In E-AT2s, by contrast, there was an increase in the percentage of mutated *Fgfr2b* from day 7 to day 14 (from 61 to 79%), indicating that continuous deletion of the *Fgfr2b* allele in AT2 cells occurred. This result supports the amplification of E-IAAPs containing wild-type *Fgfr2b* and the continuous deletion of *Fgfr2b* in E-AT2s. However, the molecular mechanisms involved in the expansion of the E-IAAPs with the wild-type *Fgfr2b* allele are still unclear. One possibility is that the previously described low level of *Sftpc* (which should translate into a lower level of Cre recombinase), associated with the closed chromatin configuration in IAAPs, renders difficult the efficient recombination of the exon 8 of the *Fgfr2b* locus in these cells.

### Reduction of tdTomato^Pos^ cells along with enhanced apoptosis and proliferation in Exp. *Fgfr2b-cKO*

**Fig. 4:**
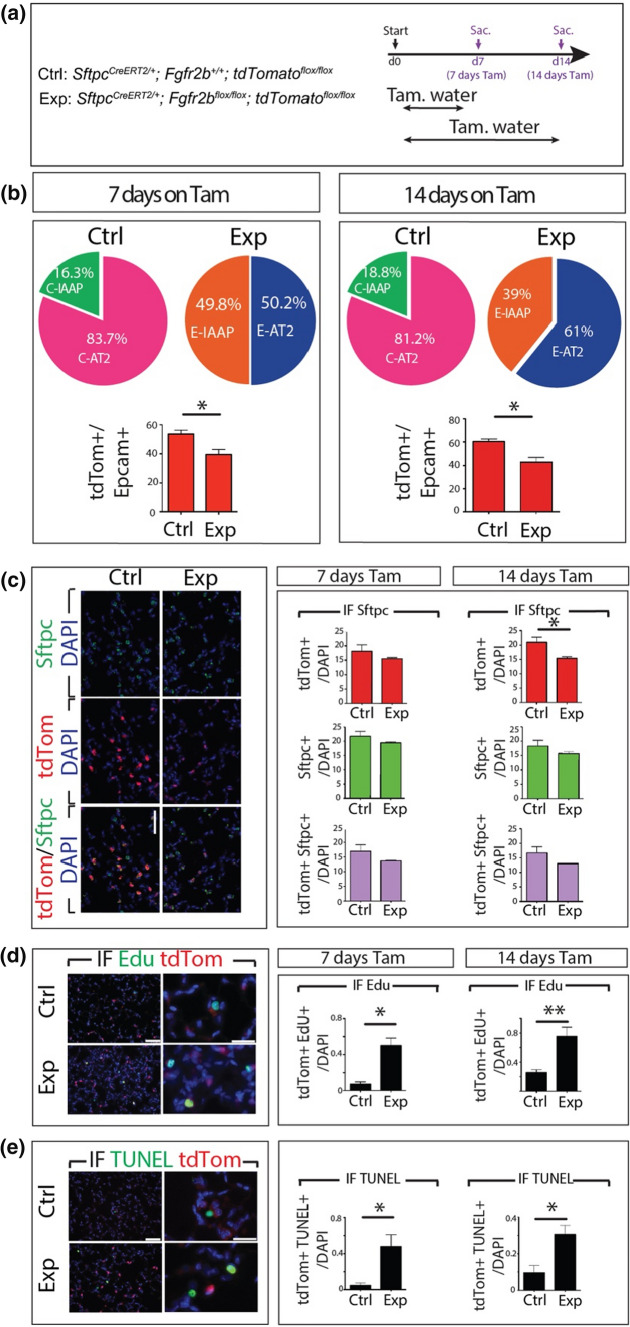
Reduction of tdTomato^Pos^ cells along with enhanced apoptosis and proliferation in *Fgfr2b-*cKO. **a** Tamoxifen treatment timeline of *Sftpc*^*CreERT2/*+^; *Fgfr2b*^+*/*+^; *tdTomato*^*flox/flox*^ and *Sftpc*^*CreERT2/*+^; *Fgfr2b*^*flox/flox*^; *tdTomato*^*flox/flox*^ mice. **b** Flow cytometry analysis of the percentage of tdTomato^Pos^ cells in Ctrl. and Exp. on day 7 and day 14. Note the expansion of the E-IAAPs as well as the decrease in tdTom+/Epcam+ in Exp. lungs. **c** Representative Sftpc immunofluorescence staining (Scale bar: 50 μm). Quantification of tdTomato+ and Sftpc+ single positive and tdTomato+ Sftpc+ double-positive cells at day 7 and day 14 (*n* = 4). **d** Representative EdU staining (Scale bar: 50 μm) and quantification of tdTomato+ Edu+ cells at day 7 and day 14 (*n* = 4). **e** Representative TUNEL staining and quantification of tdTomato+ TUNEL+ cells on day 7 and day 14. The data are presented as mean values ± SEM. *p < 0.05, **p < 0.01, ***p < 0.001

FACS analysis of the percentage of tdTomato^Pos^ over Epcam^Pos^ in Ctrl. and *Fgfr2b-cKO* indicates reduction of tdTomato-labeled cells following *Fgfr2b* deletion on day 7 and day 14 (Fig. 4a, b). Quantification of tdTom^Pos^ cells over total (DAPI^Pos^) cells on sections supports this result (Fig. 4c). In addition, Sftpc IF staining of Ctrl. and Exp. lungs was performed and quantified (Fig. 4c). The results indicate a trend towards a decrease of Sftpc^Pos^ tdTom^Pos^ over total cells in *Fgfr2b-cKO* compared to Ctrl. on days 7 and 14.

We also investigated proliferation and cell death of tdTom^Pos^ cells by immunofluorescence staining on lung sections on days 7 and 14 (Fig. 4d, e). In this context, and as previously reported [19], tomato fluorescence on sections did not distinguish between IAAPs and AT2s. On days 7 and 14, a significant increase in proliferation (Fig. 4d) and apoptosis (Fig. 4e) in tdTom^Pos^ cells was observed, suggesting that lineage-labeled subpopulations undergo apoptosis and proliferation simultaneously in Exp. lungs. These results, combined with the expansion of the IAAPs and the loss of AT2s in Exp. lungs, indicate that upon *Fgfr2b* deletion the IAAPs proliferate while the AT2s die.

To circumvent the loss of fluorescence on sections, we also isolated by FACS the E-IAAPs and E-AT2 from experimental mice (pool of *n* = 4 mice) injected with EdU one day before sacrifice (Fig. S6a). Following cytospin, the cells were analyzed for presence of Edu (Fig. S6b). Our results indicate a significant increase in EdU-positive cells in E-IAAPs compared to E-AT2s (Fig. S6c). A similar cytospin-based approach was performed for TUNEL (Fig. S7a). Our results indicated that 60% of the E-AT2 were apoptotic (Fig. S7b, c).

### The lung structure remains Intact following *Fgfr2b* deletion in the AT2 lineage

**Fig. 5:**
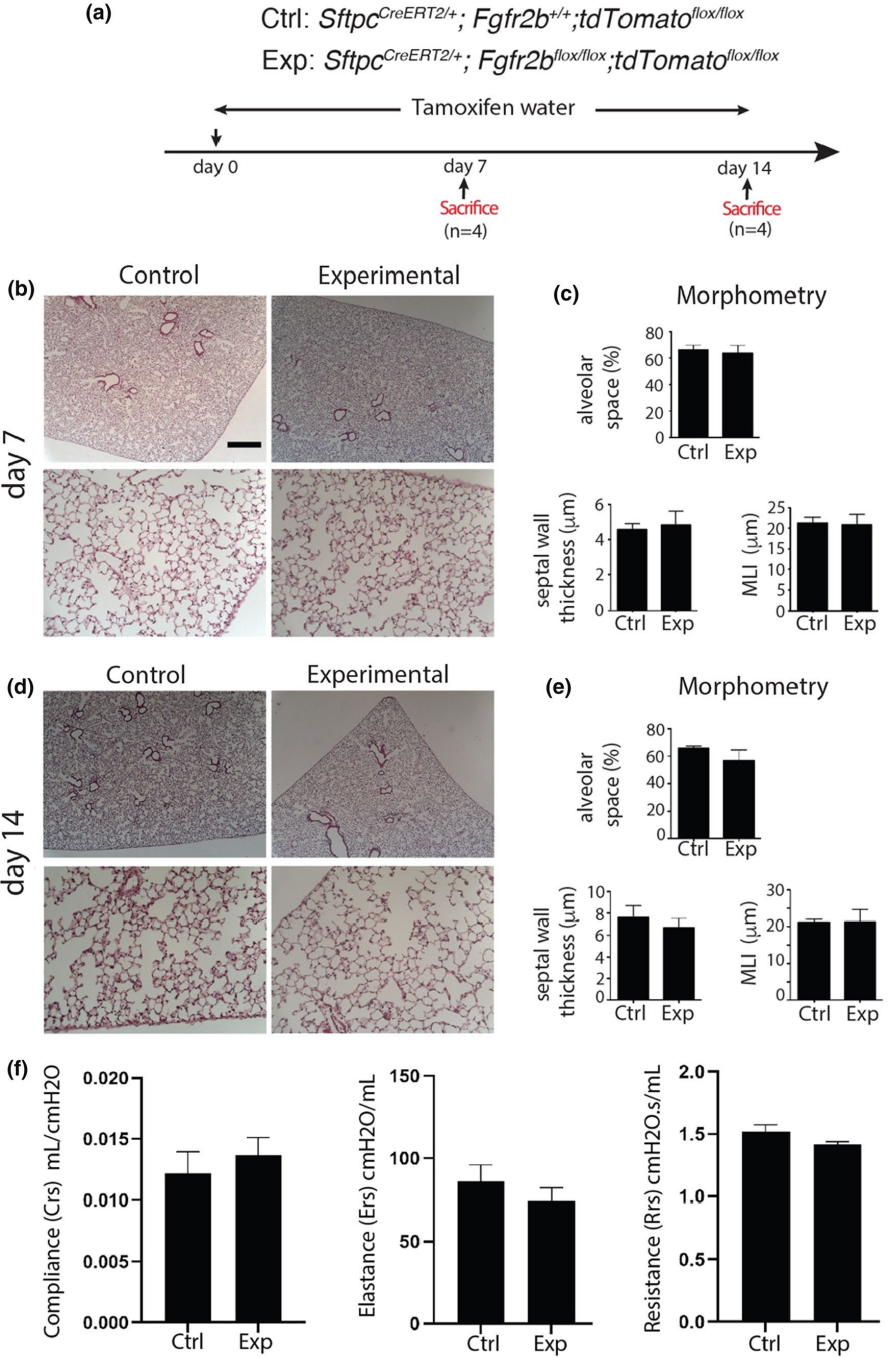
Lung structure remains Intact following *Fgfr2b* deletion. **a** Timeline of tamoxifen treatment of *Sftpc*^*CreERT2/*+^;* Fgfr2b*^+*/*+^;* tdTomato*^*flox/flox*^ and *Sftpc*^*CreERT2/*+^;* Fgfr2b*^*flox/flox*^;* tdTomato*^*flox/flox*^ mice. **b** Hematoxylin and eosin staining of the Ctrl. and the Exp. lungs at day 7 (scale bar 200 µm for lower magnification and 50 μm for higher). **c** Morphometry analysis (alveolar space, septal wall thickness, and MLI) of the Ctrl. and the Exp. lungs at day 7 (*n* = 4). **d** Hematoxylin and eosin staining of the Ctrl. and the Exp. lungs at day 14 (scale bar 200 µm for lower magnification and 50 μm for higher). **e** Morphometry analysis (alveolar space, septal wall thickness, and MLI) of Ctrl. and Exp. lungs at day 14 (*n* = 4). **f** Lung function of control and experimental mice following 2 weeks of tamoxifen exposure. The data are presented as mean values ± SEM. *p < 0.05, **p < 0.01, ***p < 0.001

To investigate whether there is a change in the lung structure after *Fgfr2b* deletion, lung morphometry analysis was performed on days 7 and 14 after tamoxifen treatment. Our results demonstrate no changes in alveolar space, septal wall thickness, or MLI in *Fgfr2b-cKO* compared to Ctrl. (Fig. 5a–e).

We also carried out morphometric analysis of control and experimental mice after 2 months and 6 months following 1-week tamoxifen treatment (Fig. S8a). No significant difference was observed between the two groups (Fig. S8b, c). In addition, measurement of lung function at day 14 after continuous tamoxifen treatment showed no significant difference between control and experimental animals (Fig. 5f).

These results suggest that the lack of abnormal lung phenotype is linked to a continuous compensatory mechanism that replenishes the mature AT2 pool. Therefore, we hypothesized that IAAPs, as immature AT2 cells, are the cells that proliferate and differentiate to mature AT2 cells.

### Deletion of *Fgfr2b* in the AT2 lineage leads to loss of self-renewal capability in mature AT2s and a gain of alveolosphere-forming potential in IAAPs

**Fig. 6:**
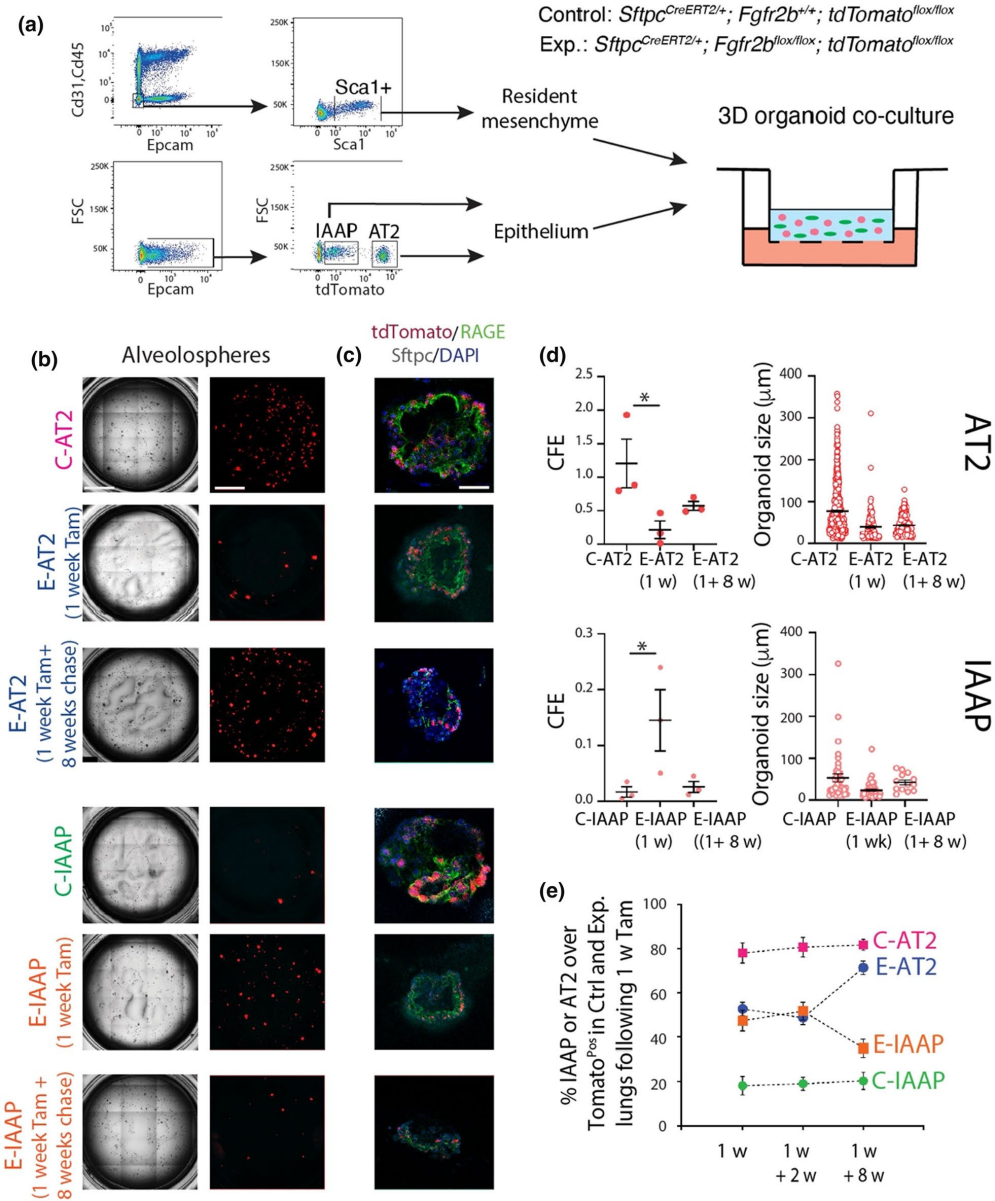
Deletion of *Fgfr2b* in the AT2 lineage leads to loss of self-renewal capability in AT2 and a gain of alveolosphere formation potential in IAAPs. **a** Representative flow cytometry shows the gating strategy of Cd31^Neg^Cd45^Neg^Epcam^Neg^population and a further selection of Sca1+ resident mesenchymal cells from C57BL/6 lungs (upper plot), as well as the selection of IAAPs and AT2s from Epcam^Pos^ population from *Sftpc*^*CreERT2/*+^;* Fgfr2b*^+*/*+^;* tdTomato*^*flox/flox*^ (lower plot). Resident mesenchymal cells were co-cultured with IAAPs and AT2s separately (*n* = 3). **b** Representative alveolospheres from AT2s and IAAPs from Ctrl. and Exp. mice (*n* = 3) (Scale bar: 100 μm). **c** Representative Sftpc and RAGE immunofluorescence staining of alveolospheres after 14 days in culture (Scale bar: 50 μm). **d** Quantification of alveolosphere size and Colony-forming unit (CFU) in AT2s and IAAPs from Ctrl. and Exp. mice (*n* = 3). **e** Percentile of AT2s and IAAPs in Ctrl. and Exp. lungs at different time points following 7 days tamoxifen treatment

To compare the proliferative capacity of IAAPs and AT2s in Ctrl. and *Fgfr2b-cKO* lungs, FACS-sorted cells were co-cultured with Cd31^Neg^Cd45^Neg^Epcam^Neg^Sca1^Pos^ resident lung mesenchymal cells according to a previously published protocol (Fig. 6a). AT2s from Ctrl. lungs behaved as *bona fide* AT2 cells as they formed alveolospheres with the expected colony-forming efficiency (Fig. 6b, c). In contrast, AT2s from *Fgfr2b-cKO* lungs demonstrated a significant decrease in alveolosphere forming capabilities compared to the corresponding Ctrl., suggesting the loss of proliferative capabilities upon *Fgfr2b* deletion (0.22% ± 0.13 vs 1.20% ± 0.36, *n* = 3) (Fig. 6d). As previously described, IAAPs from Ctrl. lungs displayed weak organoid forming capabilities, which is in line with their quiescent status [19]. Interestingly, IAAPs from *Fgfr2b-cKO* lungs showed a significant increase in alveolosphere formation, which is consistent with their transition towards the AT2 status (0.02% ± 0.01 vs 0.15% ± 0.05, *n* = 3) (Fig. 6d, e). Supporting this conclusion, we observed differential viability of FACS-isolated AT2s and IAAPs from Ctrl. and *Fgfr2b-cKO* lungs. AT2s displayed decreased viability in Exp. vs Ctrl. lungs (18.27 ± 1.64% vs 72.33 ± 5.62%, *n* = 3). In contrast, a sharp increase in viability was observed for IAAPs in Exp. vs Ctrl. lungs (72 ± 4% vs 10.17 ± 0.98%, *n* = 3) (Fig. S9). These results suggest that IAAPs in *Fgfr2b-cKO* lungs display progenitor behavior characteristics similar to mature AT2s in the Ctrl. lungs. Indeed, such progenitor-like behavior was previously suggested in vitro. Using precision-cut lung slides from *Sftpc*^*CreERT2/*+^; *Fgfr2b*^+*/*+^; *tdTom*^*flox/flox*^ mice cultured in vitro, we had demonstrated that mature AT2s were lost while IAAPs expanded. In vivo, we also showed that the IAAPs expanded following pneumonectomy [19].

### E-AT2s regain their alveolosphere-forming capabilities following a long chase period after Tamoxifen exposure

We also tested the capacity of the E-AT2s and the E-IAAPs to give rise to alveolospheres in Exp. mice exposed to Tam water for 1 week followed by a chase period of 8 weeks (Fig. 6b–d). In these conditions, a partial rescue of the capacity of the E-AT2s was observed as compared to the E-AT2s arising from animals exposed to Tam water for 1 week. On the other hand, the E-IAAPs lost their proliferative activity after such a long chase period as compared to the E-IAAPs isolated from 1-week tamoxifen treatment. The ratio of IAAPs or AT2s over the total Tom^Pos^ cells after 1-week tamoxifen water, followed by a 2-week or 8-week chase period, is represented in Fig. 6e. Our results indicate that for the 1-week and 1-week plus 2-week chase period, the percentile of E-AT2s decreases from around 80% in the controls to 50%, and the percentile of E-IAAPs increases from around 18% in the controls to 50%. However, for the 1-week tamoxifen plus 8-week chase period, these percentiles nearly returned to their control values. The complete return to normality after an 8-week chase is likely hampered by the previously reported leakiness of the *Sftpc*^*CreERT2*^ driver, which in experimental mice continuously deletes *Fgfr2b* in AT2s arising either from de novo targeted AT2s or from the IAAPs which have differentiated into AT2s (see also Fig. 8). Interestingly, such a dynamic mechanism was also observed in the context of bleomycin injury in mice, where at day 14 following bleomycin administration (at the peak of fibrosis), the AT2s decreased while the IAAPs simultaneously increased. On day 28, after fibrosis resolution had taken place, the percentile of IAAPs and AT2s had almost normalized (Zhang and Bellusci, data not shown).

### Transition of IAAPs towards AT2s in response to *Fgfr2b* deletion

**Fig. 7:**
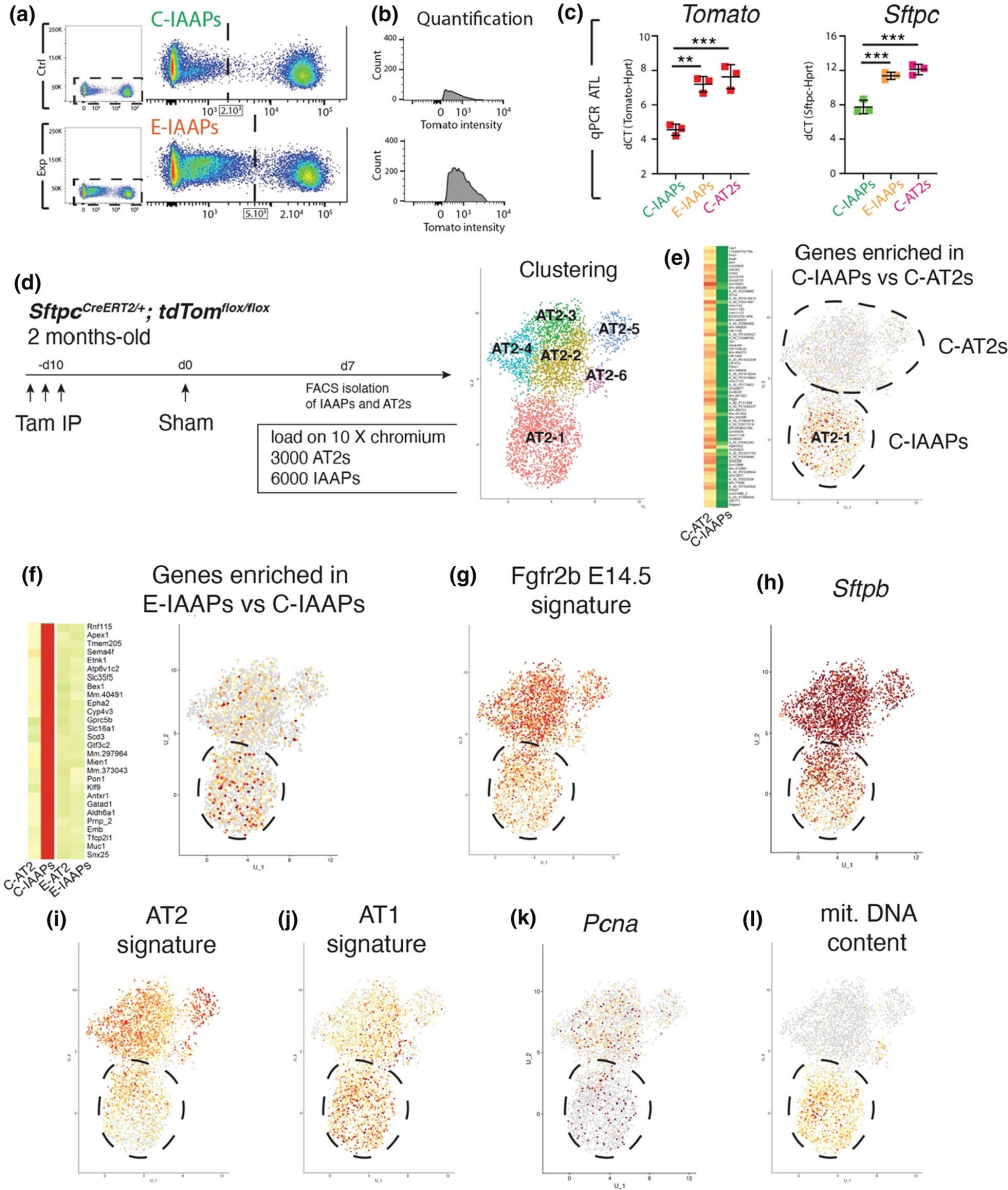
Transition of IAAPs towards AT2 in response to *Fgfr2b* deletion. **a** Representative flow cytometry analysis of tdTomato shows the expansion of IAAPs towards higher tdTomato intensity in E-IAAPs compared to C-IAAPs. **b** tdTomato intensity quantification of IAAPs in Ctrl. and *Fgfr2b-cKO* lungs. **c)** qPCR analysis of *tdTomato* expression on FACS-based sorted IAAPs. **d** scRNA-seq on FACS-isolated IAAPs and AT2s 7 days following Sham surgery. UMAP clustering indicates six main clusters. **e** Expression of genes enriched in C-IAAPs vs C-AT2s identifies the cluster AT2-1 as the C-IAAPs. **f** Expression of genes enriched in E-IAAPs vs C-IAAPS suggests that the AT2-1/IAAPs subcluster contains activated IAAPs. **g** Expression of genes representing the Fgfr2b E14.5 signature is enriched in AT2s. **h** Expression of *Sftpb* is enriched in AT2s. **i** Expression of genes representing the AT2 signature is enriched in AT2s. **j** Expression of genes representing the AT1 signature is enriched in IAAPs. **k** Expression of *Pcna* is present in both AT2s and IAAPs. **l** Expression of mitochondrial DNA genes in IAAPs. The data are presented as mean values ± SEM. *p < 0.05, **p < 0.01, ***p < 0.001

To assess how IAAPs respond to *Fgfr2b* deletion, average tdTomato intensity in IAAP cells obtained by flow cytometry in Ctrl. and *Fgfr2b-cKO* lungs was quantified (Fig. 7a, b). Our results indicate an increase in tdTomato intensity of the IAAPs in *Fgfr2b-cKO* vs Ctrl. lungs. Next, the level of expression of *Tomato* mRNA in FACS-isolated IAAPs in *Fgfr2b-cKO* vs C-IAAPs and C-AT2s in Ctrl. lungs were quantified and compared by qPCR. We found a substantial upregulation of *Tomato* expression upon *Fgfr2b* deletion (Fig. 7c), suggesting that in the *Fgfr2b-cKO* lungs, the IAAPs were transitioning towards an AT2 status. Interestingly, ATAC-seq analysis indicated more open chromatin in E-IAAPs vs C-IAAPs in the *Rosa26* locus, which contains the *tdTomato* gene (data not shown). These results are in line with the qPCR analysis of the AT2 cell differentiation marker, *Sftpc*, which indicates increased expression in E-IAAPs vs C-IAAPs and a level of expression close to that observed in C-AT2s (Fig. 7c).

### ScRNA-seq analysis of the AT2 lineage demonstrates that IAAPs and mature AT2s exist as two independent but related clusters

We used scRNA-seq to expand the profiling of IAAPs and AT2s beyond the bulk population analysis done previously. In particular, scRNA-seq enables one to better define the level of heterogeneity present in given populations (Fig. 7d–l). As we previously described, IAAPs are activated and proliferate upon pneumonectomy. For the current study, we isolated the IAAPs and AT2s on day 7 after sham or PNX. The results presented below focus only on the sham, which we considered a surrogate for Ctrl. lungs.

First, we used flow cytometry to sort IAAPs and AT2s from sham lungs (obtained from pooling these cells from 3 mice). As the C-IAAPs were described as more fragile than the C-AT2s following flow cytometry, we loaded on the 10 × chromium chip a total of 9000 cells composed of 3000 C-AT2s and 6000 C-IAAPs. Fine clustering allowed us to distinguish 6 clusters (AT2-1 to AT2-6) (Fig. 7d). Then, the lineage-labeled clusters which corresponded to the IAAPs were identified by interrogating the transcriptomic signature (arising from bulk RNAseq) obtained by comparing C-IAAPs and C-AT2s [19]. Our results indicate that the AT2-1 cluster has a high level of the IAAP signature as compared to the other clusters (Fig. 7e).

We also monitored the presence of a transcriptomic signature enriched in E-IAAPs vs C-IAAPs (Fig. 7f). The cells in the AT2-1/IAAPs cluster in our scRNA-seq displayed a higher level of this signature than in the AT2s, suggesting that the IAAPs arising from sham lungs are likely activated (Fig. 7f).

Next, we examined the Fgfr2b transcriptomic signature at E14.5 and found it to be present in the AT2-1/IAAPs cluster, albeit at a lower level compared to the AT2 cluster (Fig. 7g). Consistent with previous results, we also found that *Sftpb* expression was decreased in IAAPs vs AT2s (Fig. 7h). A similar observation was seen with the AT2 transcriptomic signature (Fig. 7i). Confirming the bulk population analysis (Fig. 2f), we found that the AT1 signature was also significantly increased in IAAPs vs AT2s (Fig. 7j). We also found that the IAAPs contained cells expressing *Pcna* and a higher level of mitochondrial content (Fig. 7k, l); thereby, supporting the previous observation that IAAPs was proliferative and metabolically active.

Interestingly, we did not find a large number of *Pd-l1* (*Cd274*) expressing cells in our data set, suggesting that either these cells did not survive in our experimental conditions (even though roughly 50% of the IAAPs should be expressing Pd-l1 both at the protein and mRNA level, Fig. S1). The alternative possibility is that these cells lost *Pd-l1* mRNA expression during scRNA-seq. In our experimental conditions, the time separating the isolation of the lungs from the loading of the cells on the 10 × Genomic chip, which is known to influence gene expression, was around 5 h.

In summary, we have demonstrated that the IAAPs and AT2s represent two transcriptionally stable and distinct populations of Sftpc^Pos^ cells. Fine clustering indicated heterogeneity in AT2s (with five subclusters). However, such heterogeneity was not detected in the IAAPs.

## Discussion

**Fig. 8:**
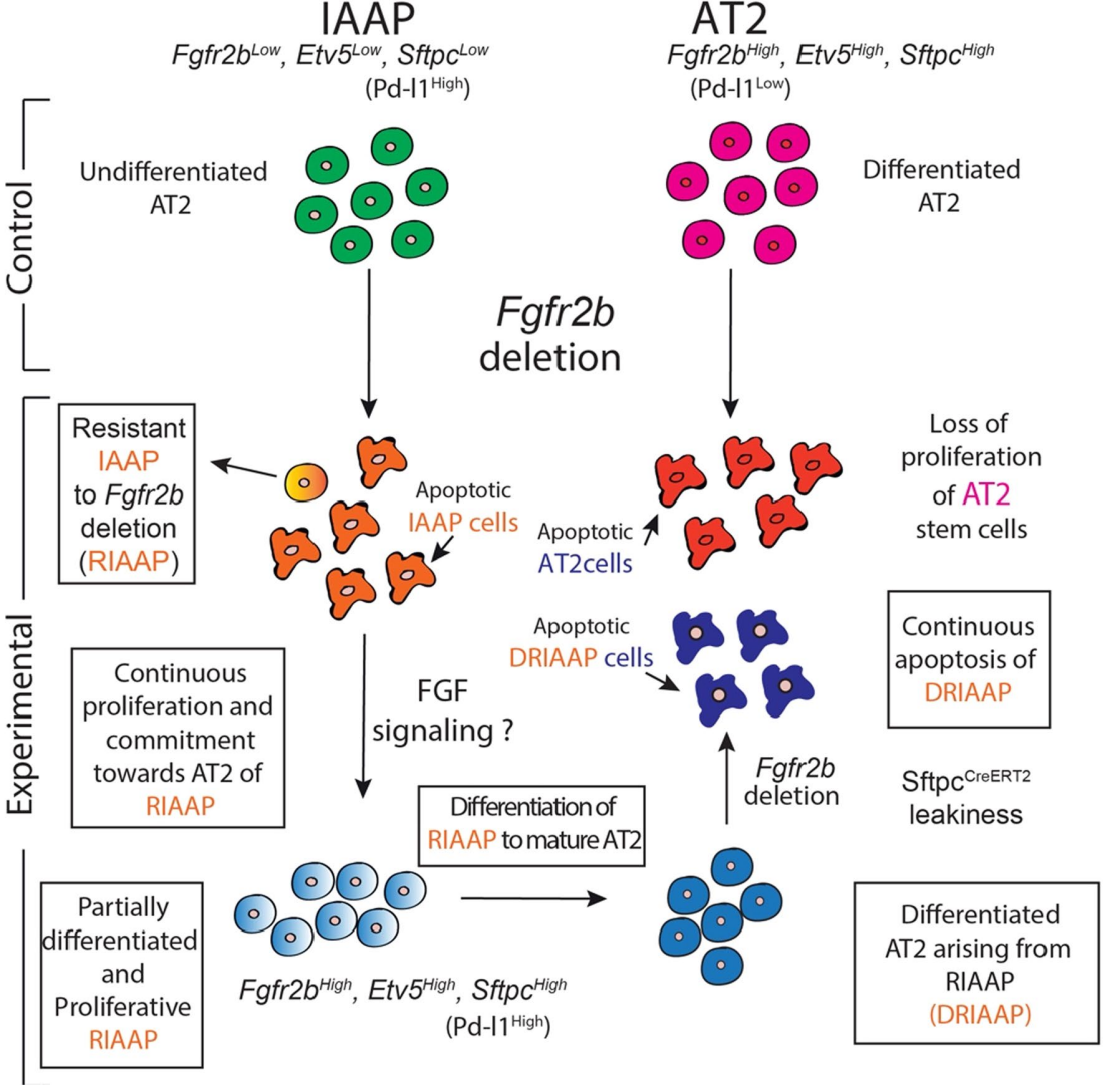
Schematic representation of characteristics and behavior of IAAP and AT2 cells in Ctrl. and *Fgfr2b-cKO* lungs. The AT2-Tom^High^ are the mature AT2s while the AT2-Tom^Low^ (IAAPs) correspond to AT2 progenitors. In the context of *Fgfr2b* deletion, both AT2s and IAAPs undergo apoptosis. However, while in the AT2 pool, this apoptotic phenotype is fully penetrant, in the IAAP pool, we observed the emergence of resistant IAAPs to *Fgfr2b* deletion (RIAAPs). These results also suggest that the IAAP pool is itself heterogeneous. The difference between RIAAPs and IAAPs and the mechanisms involved in the emergence of this resistance in RIAAPs will require further investigation. We also propose that the RIAAPs proliferate and progressively commit towards mature AT2s. We suggest that Fgfr2b signaling in these cells is likely driving this proliferation and differentiation process. Differentiated AT2s arising from RIAAPs (DRIAAPs) are then, due to the previously described leakiness of the *Sftpc*^*CreERT2*^ driver undergoing *Fgfr2b* deletion, creating a constant cycle of proliferative and apoptotic alveolar epithelial cells

The model for our study is presented in Fig. 8. Using the *Sftpc*^*CreERT2/*+^; *tdTomato*^*flox/flox*^ mice, we have previously described the existence of two distinct subpopulations of lineage-traced Sftpc^Pos^ cells based on the level of Tomato expression. The AT2-Tom^High^ represent the mature AT2 cells. On the other hand, the AT2-Tom^Low^ displayed characteristics of immature AT2 cells that could proliferate and differentiate towards mature AT2 cells in the context of pneumonectomy. These cells were proposed to represent a novel progenitor population for mature AT2 cells [19]. Owing to these characteristics, we are calling them “injury-activated alveolar progenitors” or IAAPs. In the context of *Fgfr2b* deletion, both the AT2s and IAAPs undergo apoptosis. Our ATAC-seq data supports these results showing a much higher background noise in both E-AT2s and E-IAAPs on day 7 compared to the corresponding Ctrl. Such high background noise has been associated with apoptosis [26]. In addition, IF data showed an increase in the tdTom^Pos^ TUNEL^Pos^ cells. Unfortunately, IF for Tomato does not distinguish between AT2s and IAAPs on sections, however analysis of FACS-based isolated cells followed by cytospin confirms apoptosis of E-AT2s (Fig. S7) [19]. As E-AT2 cells have lost their proliferative capabilities in the context of the alveolosphere assay, AT2s are likely the most affected cells by the loss of *Fgfr2b*. This conclusion is supported by the high expression level of the mutated transcript in E-AT2s on day 7 of tamoxifen treatment, and by the fact that in the AT2 pool, the apoptotic phenotype is fully penetrant. In the IAAP pool, we observed the emergence of lineage-labeled IAAP cells that did not display *Fgfr2b* deletion. This result suggests that in these IAAP cells, likely due to chromatin accessibility, Cre effectively acts on the *Rosa26* locus to activate Tomato expression but does not operate efficiently on the *Fgfr2b* locus to delete exon 8. These cells therefore represent transient amplifying cells with progenitor-like properties.

Interestingly, a disconnect between Tomato expression (serving as a quality control for Cre activity) and Cre-induced Diphtheria Toxin (DTA) activity in AT2 cells has been previously reported [20]. The authors used *Sftpc*^*CreERT/*+^,* R26*^*LoxP-STOP-LoxP-Tomato*^;* R26*^*LoxP-GFP-STOP-LoxP-DTA*^ to label the AT2 cells and induce at the same time the lethal expression of DTA in these cells after a single dose of tamoxifen. It was observed that lineage-labeled AT2 cells (which generally should have died due to DTA expression) proliferated clonally following AT2 killing. These data served as early evidence to demonstrate that AT2s are stem cells. To explain their results, the authors proposed that “by chance, Tamoxifen-induced recombination occurred only at the *Rosa*^*LoxP-STOP-LoxP-Tomato*^ locus in a proportion of AEC2s (AT2s), thereby lineage labeling, but not killing these AEC2s (AT2s)” [20]. A puzzling possibility in this experiment is that the lineage-labeled AT2 cells which proliferated clonally following AT2 killing arise from the lineage-labeled IAAPs.

The interpretation of these results is consistent with our published observation that in the context of precision-cut lung slices from *Sftpc*^*CreERT2/*+^; *tdTomato*^*flox/flox*^ lungs cultured in vitro, AT2s are massively killed, leaving intact the IAAPs, which then expand to become mature AT2s [19]. In the context of *Fgfr2b* deletion, rather than random recombination of one allele vs the other upon tamoxifen administration, an alternative explanation is that a subset of lineage-labeled IAAPs are, or become, resistant to *Fgfr2b* deletion, allowing them to survive. We call these cells Resistant IAAP cells to *Fgfr2b* deletion (or RIAAP cells). The mechanisms involved in the resistance in RIAAP cells will require further investigation. Novel mechanisms are likely at play as this observation is not compatible with a simple difference in chromatin opening, restricting, for example, the accessibility of the *Fgfr2b* locus. A closed chromatin configuration for the *Fgfr2* locus would expect to hamper both the recombination of the *exon 8* and the expression of *Fgfr2b* itself. As *Fgfr2b* expression is, on the contrary, increased in RIAAPs (i.e. E-IAAPs) vs C-IAAPs (see Fig. 1h), it is clear that this primary epigenetic mechanism is not sufficient to explain our results. These results also suggest that the IAAP pool is itself heterogeneous, and the difference between surviving RIAAPs and dying IAAPs will need further clarification. We also propose that the RIAAPs proliferate and become progressively committed to mature AT2s. Based on the increased expression of *Fgfr2b* and *Etv5* and the AT2 differentiation markers, we propose that Fgfr2b signaling in these cells is likely driving the proliferation and differentiation process.

Further experiments will have to be carried out to identify the Fgf ligand, likely Fgf7 or Fgf10, driving these processes. These differentiated AT2s arising from RIAAPs (called DRIAAPs) are then, due to the previously described leakiness of the *Sftpc*^*CreERT2*^ driver [19], themselves subject to *Fgfr2b* deletion, thereby creating a constant cycle of proliferative and apoptotic alveolar epithelial cells contributing to AT2 homeostasis. It is also essential to consider that non-lineage labeled AT2s are still present in experimental lungs. In our conditions, our labeling efficiency of AT2 cells is around 77% [19]. Therefore, it is possible that in the E-AT2 pool, there is a mixture of cells arising not only from E-IAAPs, but also from cells arising de novo from non-lineage labeled AT2s undergoing Cre-based recombination in a tamoxifen independent manner. Indeed, the leakiness of the Cre in the *Sftpc*^*CreERT2*^ line used for our study is relatively high and gives rise to around 5% of Tom^Pos^ cells/total cells labeled in mice exposed to water as compared to 25% in the context of tamoxifen water [19].

The long-term consequences of this new equilibrium are still unclear. In addition, how different are the DRIAAPs from *bona fide* AT2s is still unknown. The overall effect of such a process triggered by *Fgfr2b* deletion in AT2s and IAAPs is a zero-sum game preventing the appearance of a deleterious, emphysematous-like phenotype.

It was previously reported that mutant mice with specific deletion of *Fgfr2* in AT2 cells were less prone to repair after injury, displayed enhanced mortality, and had reduced AT2 cells overall. In these mice, during homeostasis, *Fgfr2* deletion resulted in increased airspace and collagen deposition, as well as a reduced number of AT2 cells [29]. These findings support our result that Fgfr2 is essential for AT2 maintenance. Our work is in line with earlier research investigating the consequences of the loss of *Etv5* in AT2 cells during homeostasis and repair after bleomycin-induced lung injury, which showed that Etv5 is required to maintain AT2 cells [30]. Upon *Etv5* deletion, AT2s transdifferentiated to AT1s. Furthermore, the repair process of the epithelium after lung injury was impaired, resulting in fewer AT2 cells altogether. Etv5 is known to be regulated by Fgfr2b signaling during lung development [31], and it has been suggested that Etv5 in AT2 cells is controlled by Ras-mediated ERK signaling [30]. However, a more recent paper has also reported on the inactivation of *Fgfr2* in AT2s using the *Sftpc*^*CreERT2*^ driver line [14]. This study concluded that Fgfr2 signaling, while preventing the differentiation of AT2s towards the AT1 lineage during alveologenesis, *is dispensable* during homeostasis in the adult. This latter conclusion is at odds with our findings.

In our conditions, loss of Fgfr2b signaling in AT2s leads to a significant decrease in their proliferative capacity using the alveolosphere assay. This result was not observed by Liberti et al. [14]. A methodological difference that could explain this discrepancy in results is that a different tamoxifen regimen was used. While we primarily studied the impact of *Fgfr2b* deletion on day 7 from the start of tamoxifen delivery via water, Liberti et al. treated the experimental adult mice by oral gavage with tamoxifen for three consecutive days followed by 2 weeks washout period. We have also analyzed the experimental lungs after a 2-week or 8-week chase period (Fig. S10).

We observed decreased ratio of Tom^Pos^ cells over EpCAM as well as decreased ratio of Tom^Pos^/DAPI, Sftpc^Pos^/DAPI and Tom^Pos^Sftpc^Pos^/DAPI in Exp. vs Ctrl. lung after a 2-week chase period (Fig. S10b, c). In contrast, no significant difference in these parameters was observed between Exp. vs Ctrl. lung after an 8-week chase period (Fig. S10e, f). Interestingly, RT-PCR after the 2-week chase period shows that the WT transcript is dominant in both the E-AT2s and E-IAAPs (Fig. S10a), supporting a progressive normalization of these parameters over time following *Fgfr2b* deletion and indicating the establishment of another homeostatic equilibrium This conclusion is supported by the quantification of proliferation and apoptosis, which is no longer significant between Exp. and Ctrl. lungs after 8-weeks chase period (Fig. S10d).

In addition, the difference between our conditions and the ones from Liberti et al. could be due to different leakiness levels of the *Sftpc*^*CreERT2*^ driver used. Interestingly, Liberti et al. also reported, using IF, an increase in Edu^Pos^ lineage-traced cells in the Exp. vs Ctrl. lungs without providing a clear explanation for this controversial result. Fgfr2b signaling is known to positively control proliferation and/or survival. However, to our knowledge, it does not inhibit proliferation per se. The interpretation for this puzzling result is now clear if we consider the proliferative lineage-traced E-IAAPs and the new homeostatic equilibrium present in the experimental lungs.

We have previously reported that C-IAAPs express Pd-l1 [19]. We found similar results for E-IAAPs (Fig. S1). In the context of cancer, PD-L1 expressed by some human cancer cells binds to PD1, a checkpoint protein expressed by T cells to prevent the immune cells from attacking them, allowing the cancer cells to escape the immune aggression. These cells usually display enhanced self-renewal capabilities and are considered cancer stem cells [21, 32–34]. A similar concept is emerging in the context of the IAAPs with their capacity to escape the harmful consequences of *Fgfr2b* inactivation. Although these escaping properties may be beneficial in the context of lung injury, future research should also focus on examining their role in the context of cancer. Designing dual-labeling systems such as the Dre/Rox and Cre/LoxP systems [35] under the control of a *Sftpc* and *Pd-l1* promoter appears to be a promising strategy to specifically target the IAAPs and examine their precise contribution to the AT2 lineage in the context of repair after injury, regeneration, or even in cancer.

In conclusion, we have identified IAAPs as a potentially novel population of AT2 progenitors necessary for alveolar repair after massive injury to mature AT2s. Understanding how IAAPs are activated to proliferate and differentiate into mature AT2s will be critical for designing efficient strategies to treat debilitating lung diseases.

## Supplementary Information

Below is the link to the electronic supplementary material.

Supplementary file1 (PDF 7141 KB)

## Data Availability

The scRNA-seq data are currently been deposited in GEO (accession number GSE199112). Genearrays data have been been deposited in GEO (accession number GSE162588).
