# Supplementary material for: Fgfr2b signaling is essential for the maintenance of the alveolar epithelial type 2 lineage during lung homeostasis in mice
Source: Cell Mol Life Sci. 2022 May 19;79(6):302. doi: 10.1007/s00018-022-04327-w (PMC9120111; doi:10.1007/s00018-022-04327-w)
Supplement: Supplementary file 1 — Supplementary file1 (PDF 7141 KB) [file 18_2022_4327_MOESM1_ESM.pdf]

Fgfr2b signaling is essential for the maintenance of the alveolar epithelial type 2 lineage during lung homeostasis in mice

Negah Ahmadvand[1], Arun Lingampally[1], Farhad Khosravi[2], Ana Ivonne Vazquez-Armendariz1,[3], Stefano Rivetti[1], Matthew R. Jones[1], Jochen Wilhelm[1,3], Susanne Herold[1,3], Guillermo Barreto[4], Janine Koepke[1], Christos Samakovlis[1], Gianni Carraro[5], Jin-San Zhang[6], Denise Al Alam[7], Saverio Bellusci[1,3 #]

Figure S1_Ahmadvand et al

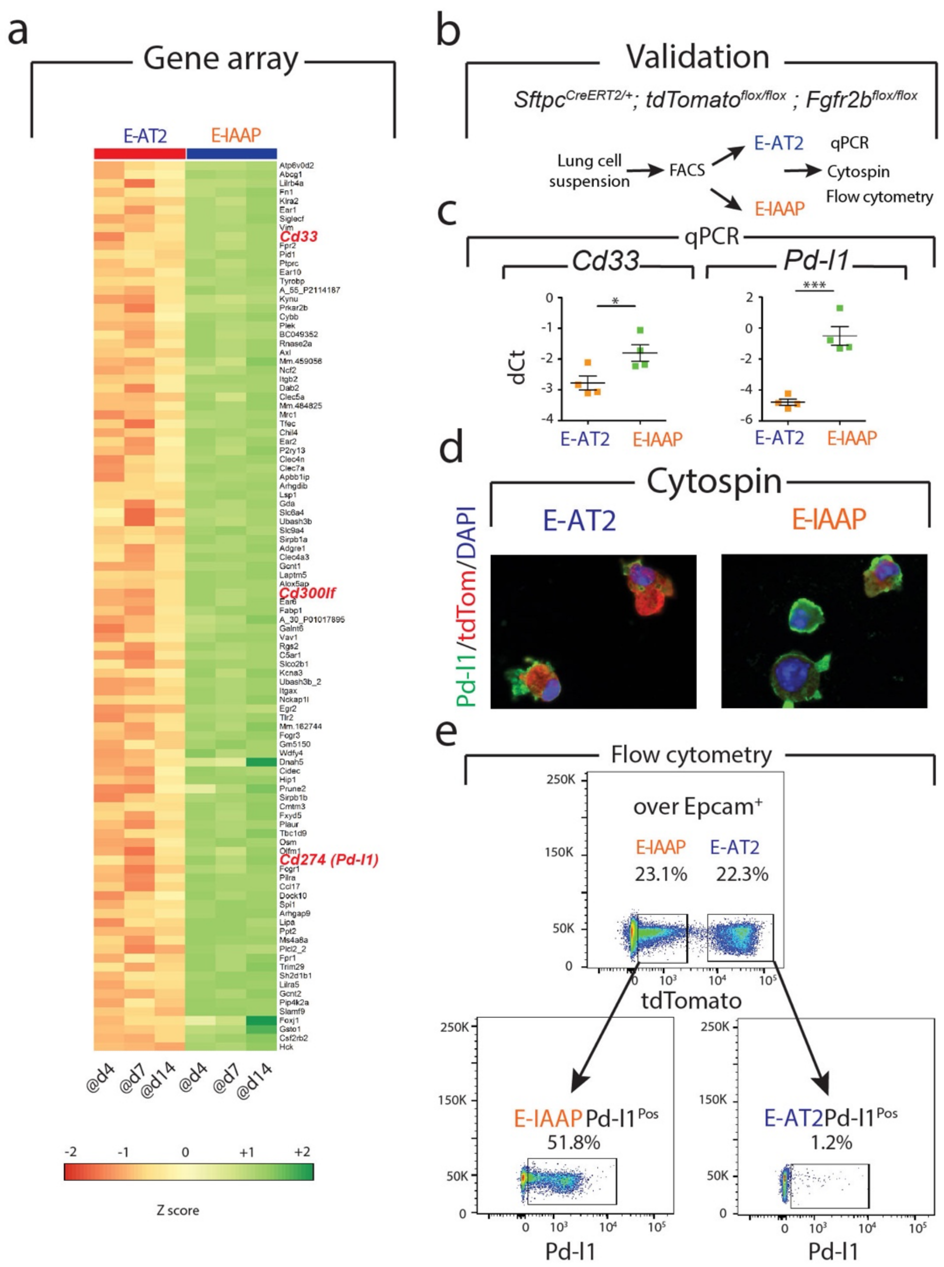

Fig. S1 Enrichment of Pd-l1 expression in E-IAAPs vs E-AT2s. **a)** Gene array for E-AT2s and E-IAAPs collected on day 4, 7 and 14 during the tamoxifen treatment. Note that *Cd33*, *Cd300lf* and *Cd274* (aka Pd-l1) are increased in E-IAAPs vs E-AT2s. **b)** Validation of these results by qPCR, cytospin and flow cytometry. **c)** qPCR indicates that *Cd33* and *Pd-l1* are enriched in E-IAAPs. **d)** Cystospin followed by IF for Pd-l1 indicates enrichment in Pd-l1 protein expression in E-IAAPs. **e)** Flow cytometry for E-IAAPs and E-AT2s followed by detection of Pd-l1 confirms that Pd-l1 is expressed chiefly in E-IAAPs.

Figure S2_ Ahmadvand et al

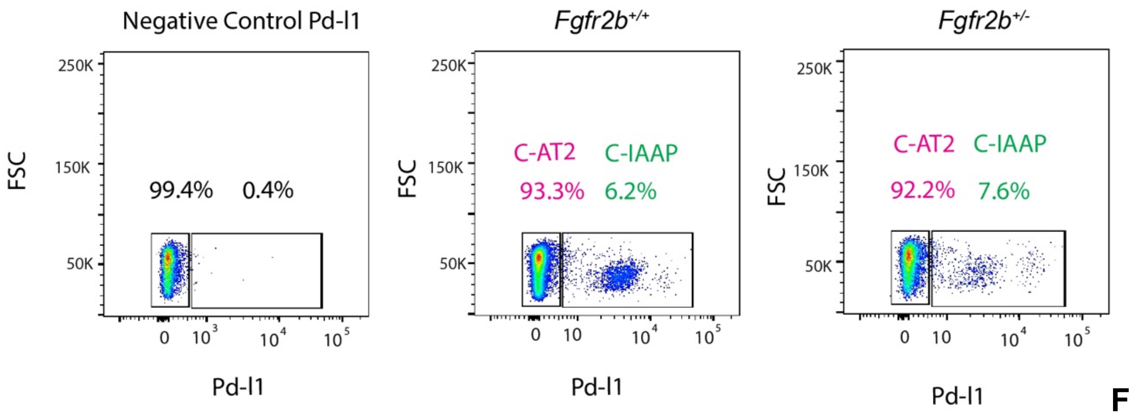

Fig. S2 Fgfr2b is haplosufficient in AT2s. FACS-based approach using antibodies against Pd-l1 to isolate Pdl1^{Neg} AT2s and Pdl1^{Pos} IAAPs in *Fgfr2b*$^{+/+}$ and *Fgfr2b*$^{+/-}$ lungs.

Figure S3_Ahmadvand et al

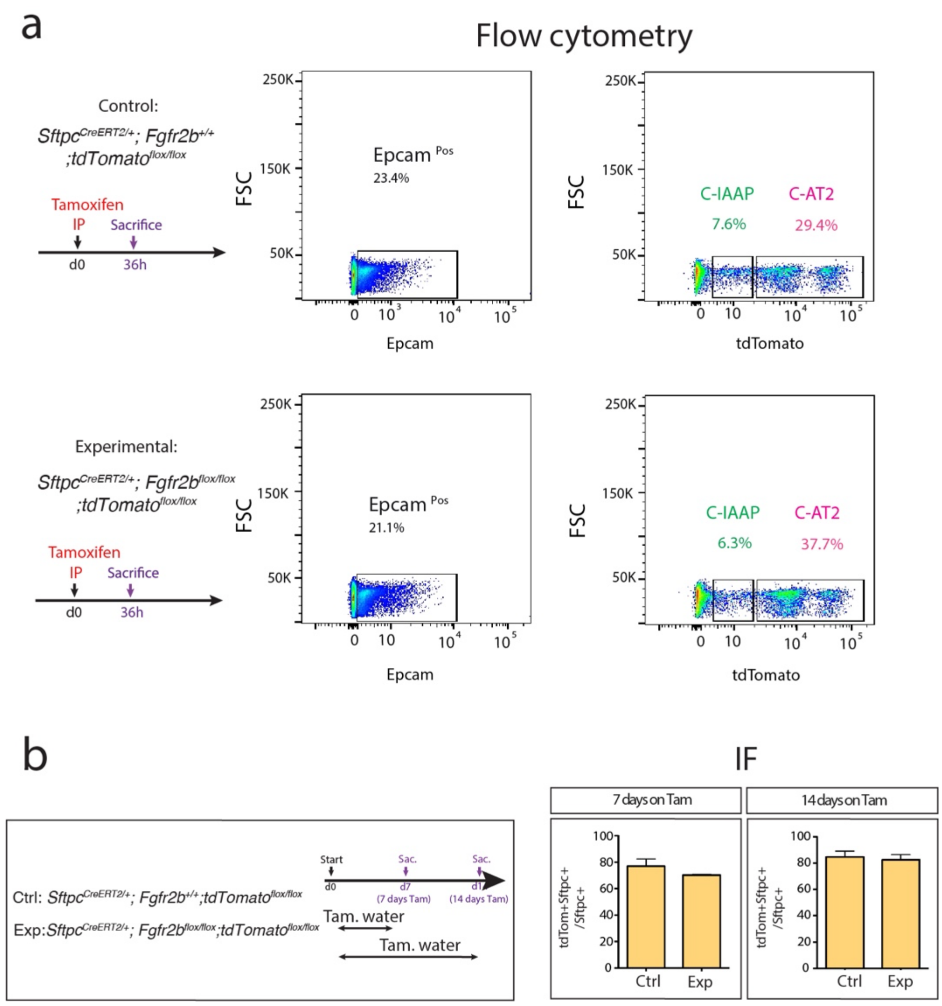

Fig. S3 Recombination efficiency in the IAAPs and AT2s in Exp. vs Ctrl. Lungs. a) Ctrl. and Exp. lungs were analyzed 36 hours after a single dose of Tam IP. FACS analysis was carried out to quantify the abundance of IAAPs and AT2s (out of Epcam) in Ctrl. and Exp. lungs. **b)** Recombination efficiency in one-week and two-week tamoxifen treated animals. Quantification of the % of $Tom^{Pos}Sftpc^{Pos}/Sftpc^{Pos}$ by IF.

Figure S4_Ahmadvand et al

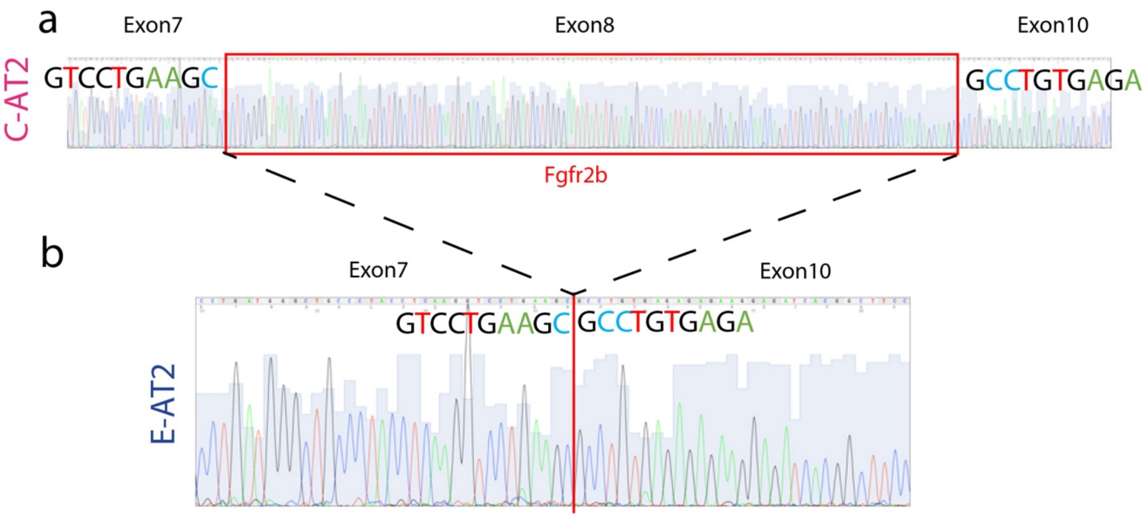

Fig. S4 Sequencing of the WT and mutant Fgr2b transcripts. a) Sequencing of WT amplified cDNA (340 pb) indicating the presence of Exon 8. **b)** Sequencing of mutant amplified cDNA (195 pb) indicating the deletion of Exon 8.

Figure S5_Ahmadvand et al

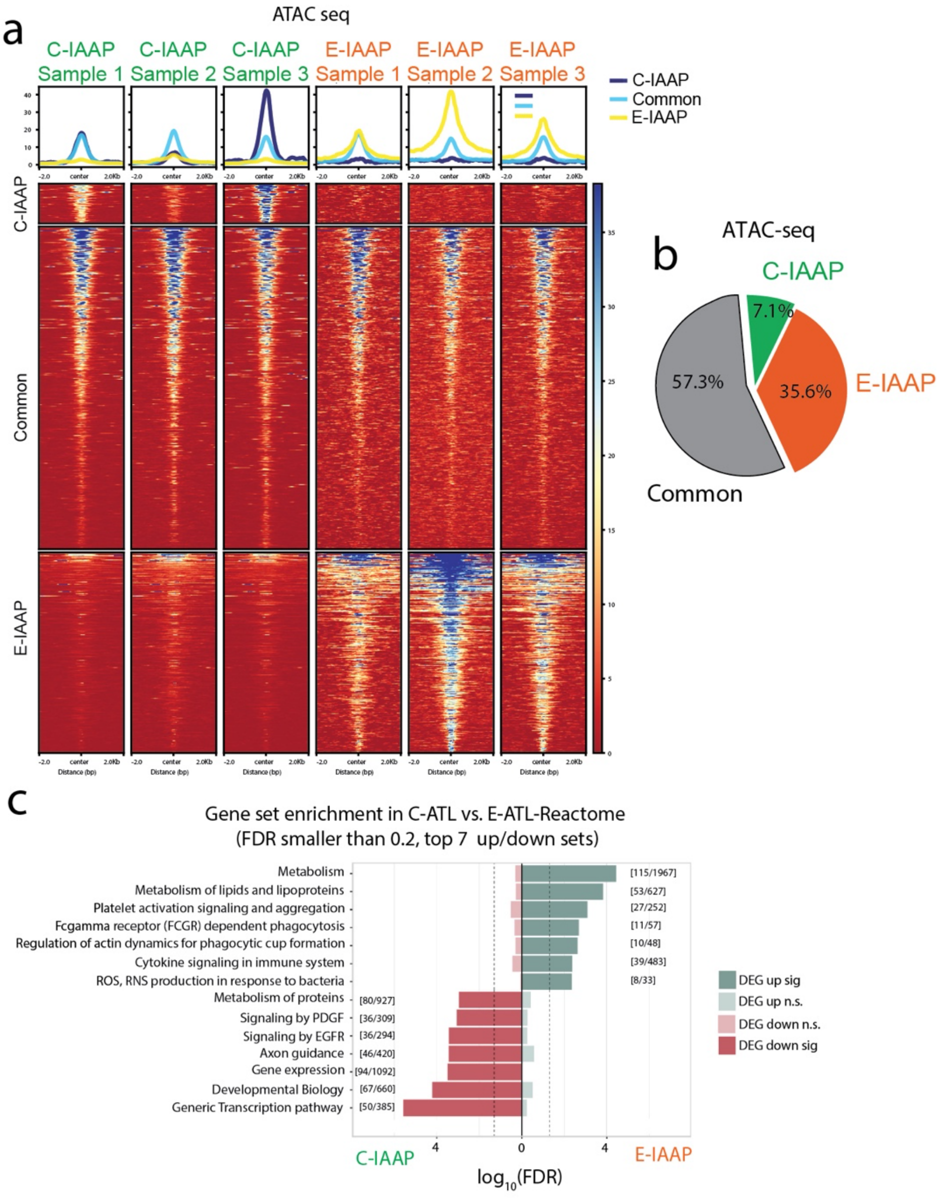

Fig. S5 ATAC-seq analysis of E-IAAPs and C-IAAPs suggest that IAAPs are activated upon *Fgfr2b* deletion. a-b) Coverage heat maps of C-IAAPs and E-IAAPs, displaying genome-wide regions of differential open chromatin peaks in E-IAAPs vs C-IAAPs. C-IAAP chromatin is less open and transcriptionally less active compared to E-IAAPs. ATAC-seq analysis of peaks based on the cutoffs shows 56 up-regulated genes in C-IAAPs (FDR < 0.05, I log_2(FC) > 0.585, base Mean > 20) , 455 up-regulated genes in E-IAAPs (FDR < 0.05, log_2(FC) > 0.585, base Mean > 20) and 455 non-regulated genes (base Mean > 20, FDR > 0.5, log_2(FC) between -0.15 and 0.15) which means 7.1% and 35.6% of the genome is differently accessible in C-IAAPs and E-IAAPs, respectively. **c**) Analysis of peaks obtained in the ATAC-seq experiment for E-IAAPs and C-IAAPs using Kobas for the Reactome database. Peaks overlapping gene body or near the transcription starting site of

genes were annotated to the corresponding genes. All annotated peaks were split into lists of genes that display more open chromatin in E-IAAPs or C-IAAPs using DESeq2 on unified peak regions. Observed significance was adjusted by Benjamini-Hochberg correction for multiple tests (FDR). The resulting lists were used as input for Kobas to search for enriched terms in different databases. The top seven terms were chosen by significance (FDR < 0.2). Results indicate that the term ‘Metabolism’ is highly enriched in E-IAAPs, indicating that the chromatin of E-IAAPs is more accessible in loci of genes (gene body or promoter) associated with metabolism. Higher accessibility is associated with more transcriptional activity. Numbers in brackets display the number of identified genes / total number of genes for term in the database. DEG: Differentially expressed genes. Between brackets [ ]: Genes found/total genes in term.

Figure S6_Ahmadvand et al

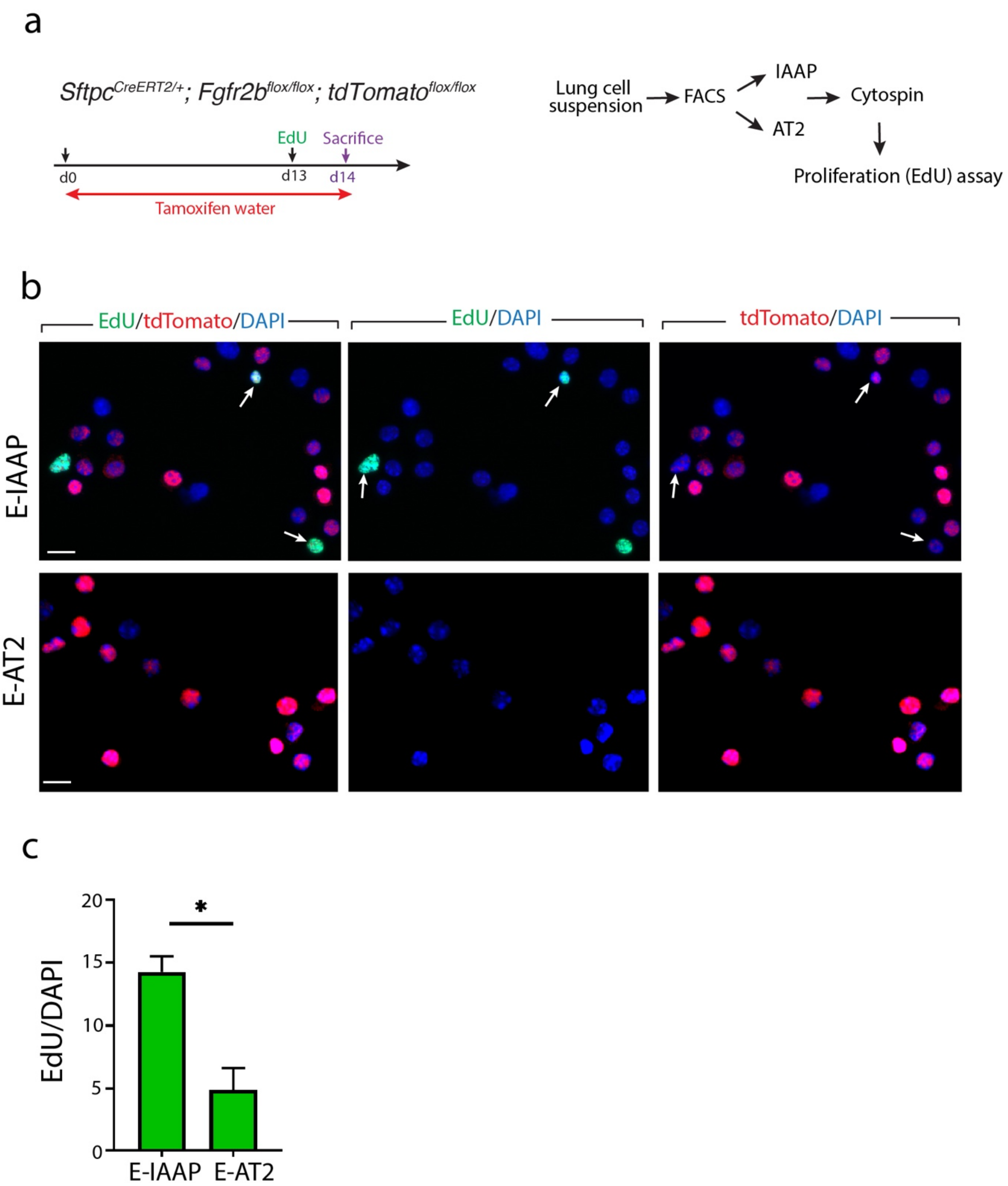

Fig. S6 Increased proliferation in E-IAAPs versus E-AT2. a) Timeline of tamoxifen treatment of *Sftpc*$^{CreERT2/+}$; *Fgfr2b*$^{flox/flox}$; *tdTom*$^{flox/flox}$ experimental mice (n=4). Sacrifice of the mice was carried out at d14 following EdU injection at day 13. **b)** Detection of EdU and Tomato by IF following cytospin of E-IAAPs and E-AT2s. **c)** Corresponding quantification of EdU labeling. Scale bar: 50µm.

Figure S7_Ahmadvand et al

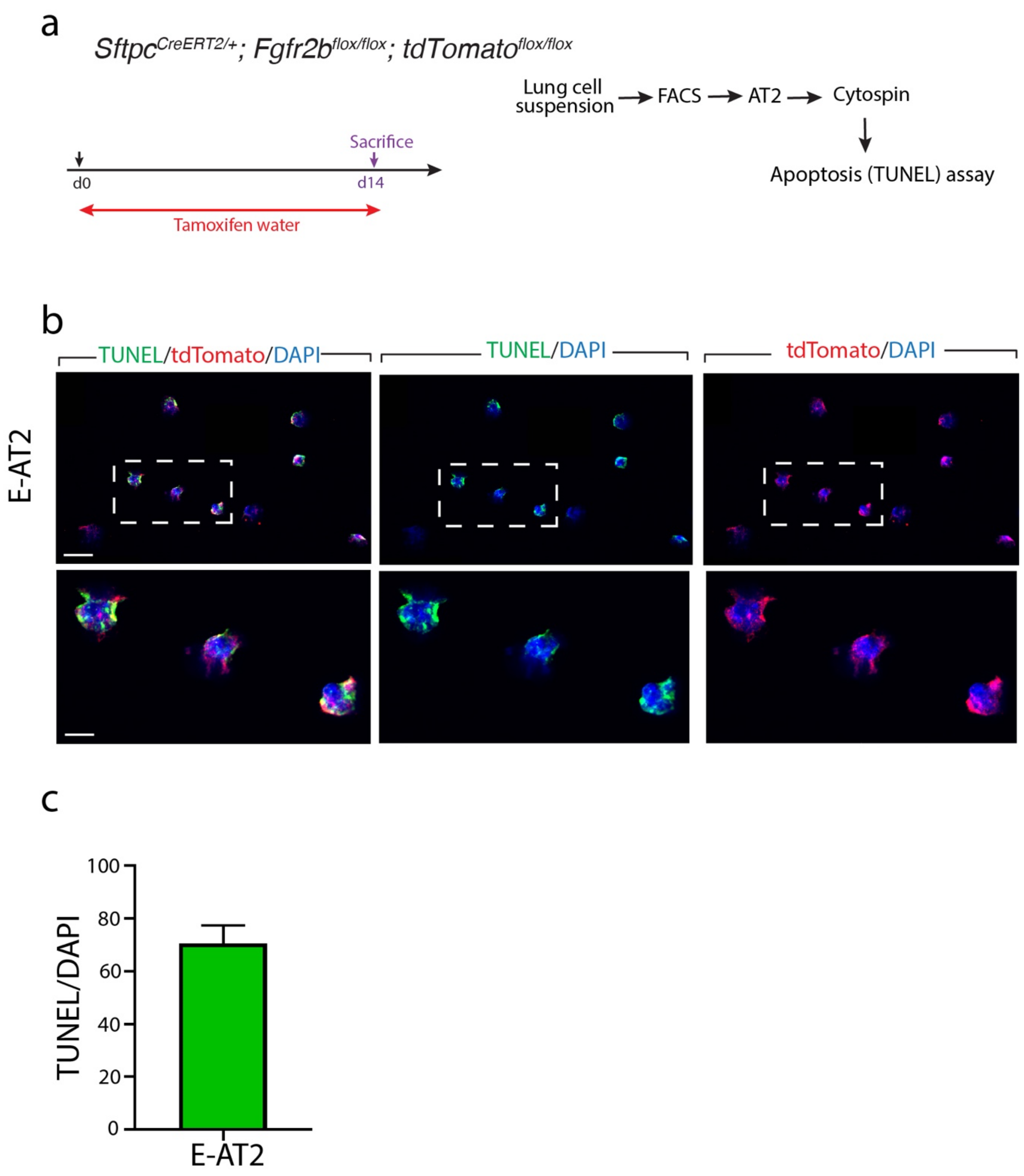

Fig. S7 Increased apoptosis of the E-AT2s. a) Timeline of tamoxifen treatment of *Sftpc*$^{CreERT2/+}$*; Fgfr2b*$^{flox/flox}$*; tdTom*$^{flox/flox}$ experimental mice (n=4). Sacrifice of the mice was carried out at d14. b) Detection of TUNEL and Tomato by IF following cytospin of E-AT2s. **c)** Corresponding quantification of TUNEL labeling. Scale bar: 50µm.

Figure S8 _Ahmadvand et al

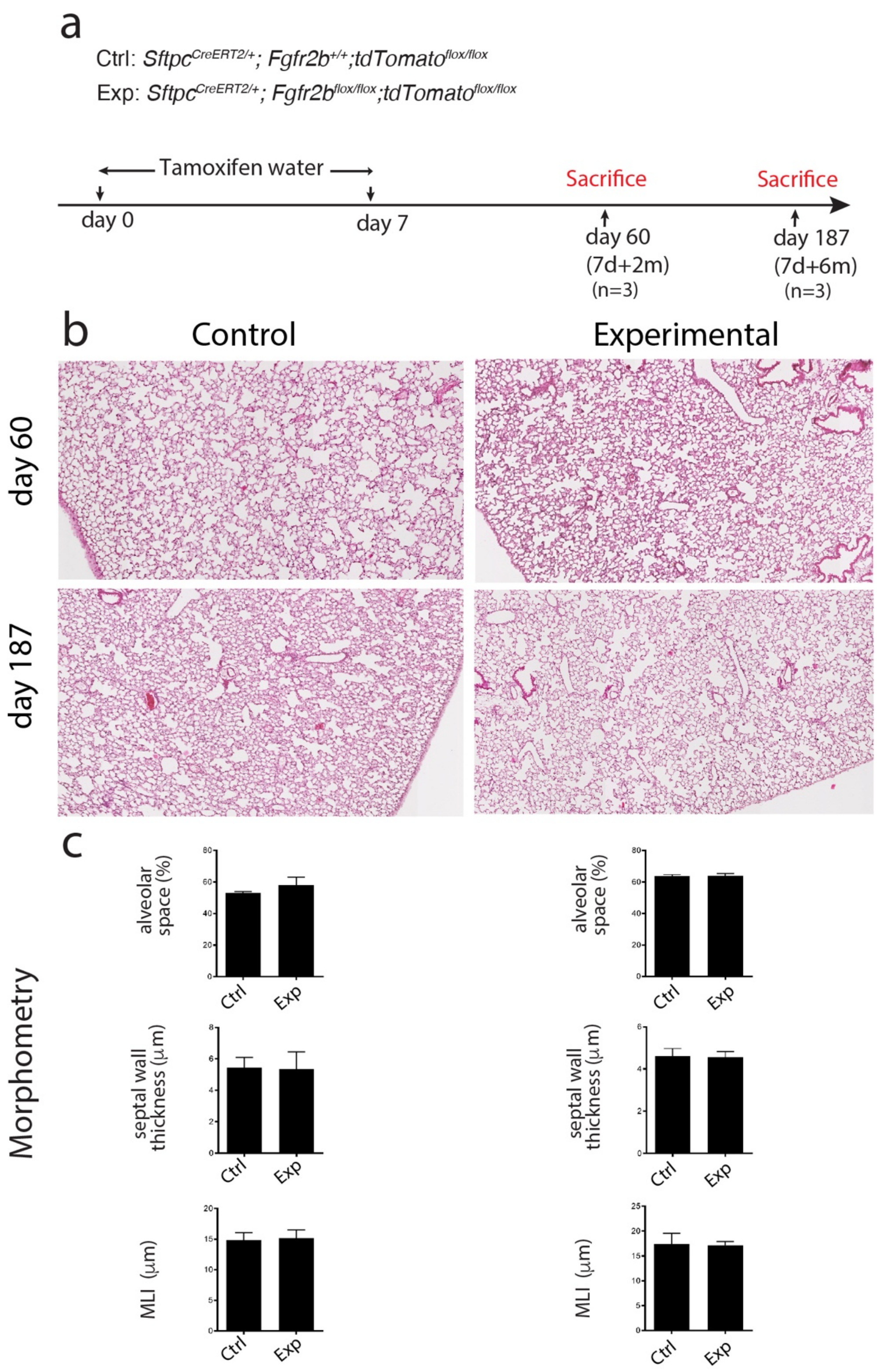

Fig. S8 Normal morphometry of experimental lungs after 2 and 6 months. a) Timeline of tamoxifen treatment of *Sftpc*$^{CreERT2/+}$; *Fgfr2b*$^{+/+}$; *tdTomato*$^{flox/flox}$ and

Sftpc$^{CreERT2/+}$*; Fgfr2b*$^{flox/flox}$*; tdTomato*$^{flox/flox}$ mice. **b)** Hematoxylin and eosin staining of the Ctrl. and the Exp. lungs at day 60 and day 187 (scale bar 200 µm) **c)** Corresponding morphometry analysis (alveolar space, septal wall thickness, and MLI) of the Ctrl. and the Exp. lungs (n=4).

Figure S9_Ahmadvand et al

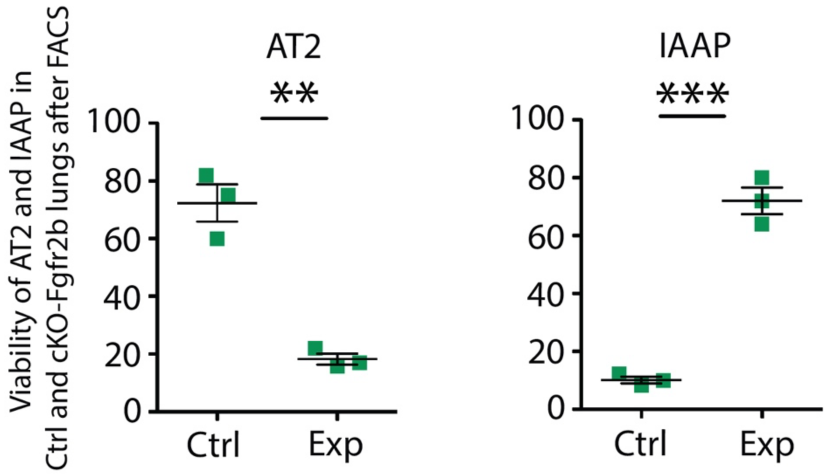

Fig. S9 Viability of IAAPs and AT2s in Ctrl. and *Fgfr2b-cKO* lungs following FACS

Quantification of live and dead cells of Tom^{Low} and Tom^{High} by NucleoCounter following FACS isolation from Exp. compared to Ctrl. mice (n=4). Data are presented as mean values ± SEM. ∗$p < 0.05$, ∗∗$p < 0.01$, ∗∗∗$p < 0.001$

Figure S10_Ahmadvand et al

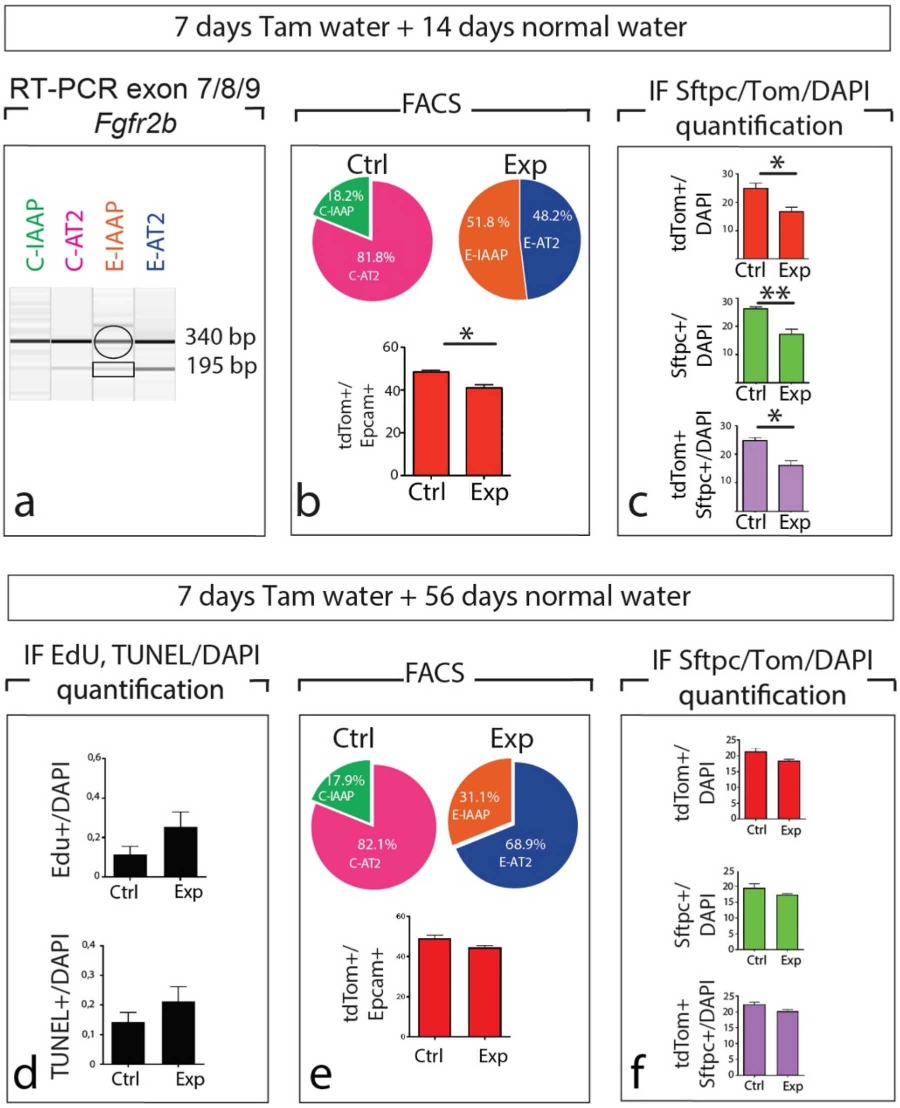

Fig. S10 Analysis of the AT2s and IAAPs in Ctrl. and Exp. Lungs. a-c) Analysis of the AT2s and IAAPs in Ctrl. and Exp. Lungs in one-week tamoxifen followed by a two-week chase period. **a**) RT-PCR for detecting WT and *Fgfr2b* mutant transcripts in FACS-based sorted C-IAAPs, C-AT2s, E-IAAPs and E-AT2s. **b**) Flow cytometry analysis indicating expansion of the E-IAAPs, as well as a global decrease in tdTom+ cells/Epcam+ in Exp. lungs. **c**) IF for tdTom+ and Sftpc+ single positive cells as well as tdTom+ Sftpc+ double-positive cells. **d-e**) Analysis of the AT2s and IAAPs in Ctrl. and Exp. lungs in one-week tamoxifen followed by an eight-week chase period. **d**) IF for Edu and TUNEL indicating a trend (non-significant) towards a residual increase in proliferation and apoptosis. **e**) Flow cytometry analysis indicating that the percentile of E-IAAPs, even though still higher than the one observed for C-IAAPs, is trending towards a normalization. Note that there is no change in the number of tdTom+/Epcam+ in Exp. and Ctrl. lungs at this time point. **f**) IF for tdTom+ and Sftpc+ single positive cells as well as tdTom+ Sftpc+ double-positive cells show no difference between Ctrl. and Exp. lungs.
